# Pattern and timing of diversification in the African freshwater fish genus *Distichodus* (Characiformes: Distichodontidae)

**DOI:** 10.1186/s12862-020-01615-6

**Published:** 2020-04-26

**Authors:** Jairo Arroyave, John S. S. Denton, Melanie L. J. Stiassny

**Affiliations:** 1 Instituto de Biología, Universidad Nacional Autónoma de México, Circuito Zona Deportiva 53, Ciudad Universitaria, 04510 Coyoacán, Ciudad de México, Mexico; 2 Department of Ichthyology, American Museum of Natural History, Central Park West at 79th Street, New York, NY 10024 USA; 3 Florida Museum of Natural History, University of Florida, Dickinson Hall, 1659 Museum Road, Gainesville, FL 32611 USA

**Keywords:** Distichodontidae, *Distichodus*, Congo Basin, Molecular phylogeny, African fishes, Geographic range evolution, Molecular dating

## Abstract

**Background:**

*Distichodus* is a clade of tropical freshwater fishes currently comprising 25 named species distributed continent-wide throughout the Nilo-Sudan and most Sub-Saharan drainages. This study investigates the phylogenetic relationships, timing of diversification, and biogeographic history of the genus from a taxonomically comprehensive mutilocus dataset analyzed using Maximum Likelihood and Bayesian methods of phylogenetic inference, coalescence-based species-tree estimation, divergence time estimation, and inference of geographic range evolution.

**Results:**

Analyses of comparative DNA sequence data in a phylogenetic context reveal the existence of two major clades of similar species-level diversity and provide support for the monophyletic status of most sampled species. Biogeographic reconstruction on a time-scaled phylogeny suggest that the origins of the genus date back to the late Oligocene and that current geographic distributions are the result of a Congo Basin origin followed by dispersal and range expansion into adjacent ichthyofaunal provinces at different times during the evolutionary history of the group.

**Conclusions:**

We present the most comprehensive phylogenetic, chronological, and biogeographic treatment yet conducted for the genus. The few instances of species paraphyly (*D. teugelsi, D. fasciolatus*) revealed by the resulting phylogenies are likely a consequence of post-divergence introgressive hybridization and/or incomplete lineage sorting due to recent speciation. Historical biogeographic findings are both in agreement and conflict with previous studies of other continent-wide African freshwater fish genera, suggesting a complex scenario for the assemblage of Africa’s continental ichthyofaunal communities.

## Background

**Fig. 1:**
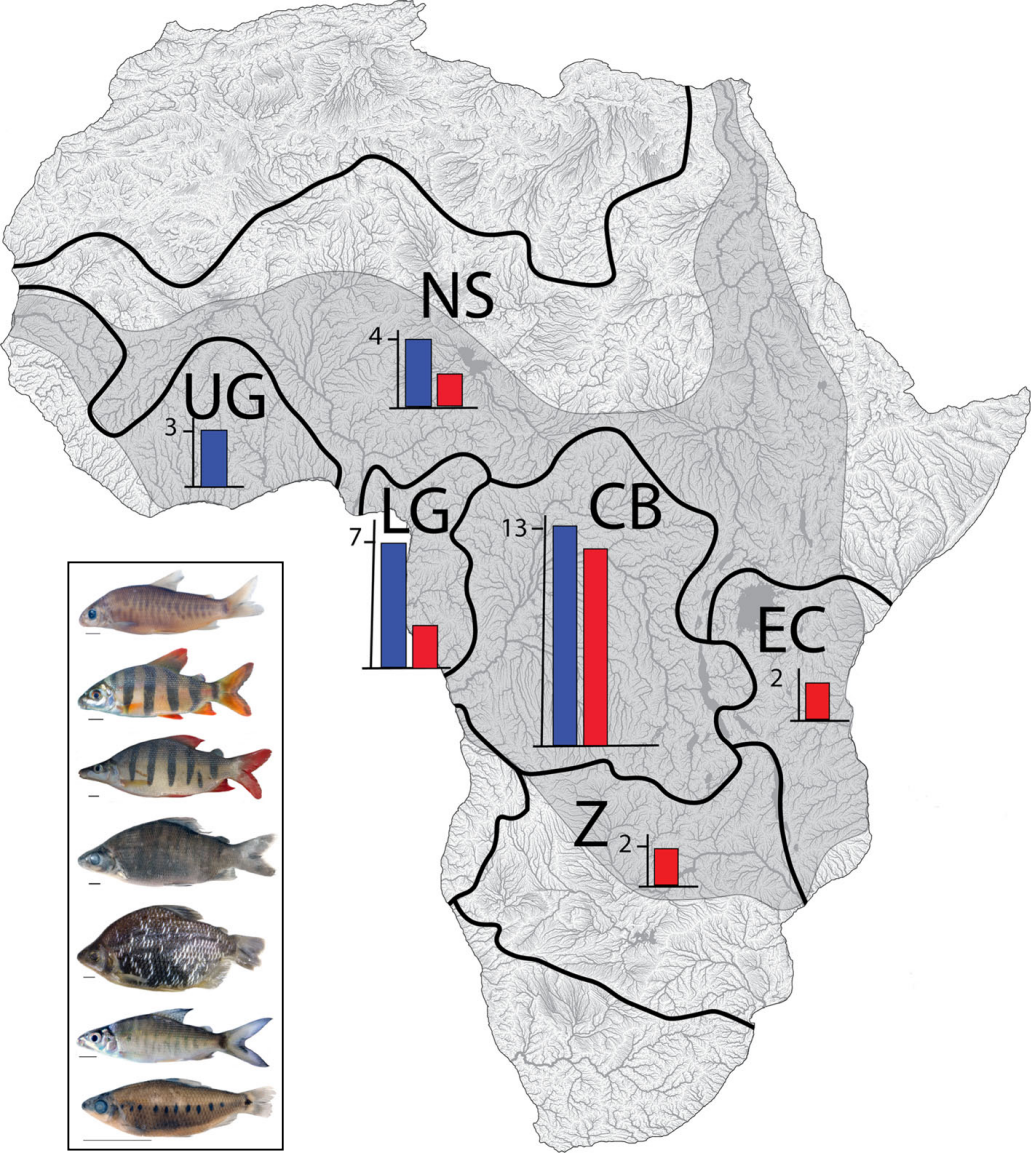
Geographic distribution and variation in external morphology of *Distichodus* species diversity. Map of Africa divided into ichthyofaunal provinces (originally defined by Roberts [1], modified by Lévêque [2], and redrawn according to new hydrological basin mapping published by FAO [3]): Congo Basin (CB), East Africa (EA), Nilo-Sudan (NS), Lower Guinea (LG), South Africa (SA), and West Africa (WA). Shaded area represents *Distichodus* extent of occurrence. Inset bar charts indicate number of *Distichodus* species present in each ichthyofaunal province: endemic (red) and total (blue) (when more than endemics). Inset frame fish photographs illustrate the extent of variation in body shape, size, and coloration in *Distichodus* species (from top to bottom: *D. hypostomatus*, *D. sexfasciatus*, *D. lussoso*, *D. antonii*, *D. affinis*, *D. shenga*, *D. decemmaculatus*)

*Distichodus*, the type genus of the endemic African characiform family Distichodontidae, is a morphologically distinctive and moderately speciose lineage of endemic African freshwater fishes. *Distichodus* species are distributed across the continent, occurring throughout the freshwaters of most of sub-Saharan Africa and the river basins of the Nilo-Sudan, with representation in six of the nine ichthyofaunal provinces of continental Africa (Fig. 1). Although general aspects of the biology of the genus are poorly documented, a few studies indicate that most species are typically diurnally active and found primarily in lentic habitats shoaling in and around grasses along vegetated river banks and swamps [4]. Most species are primarily herbivorous, feeding almost entirely on periphyton, macrophytes, and detritus [5–7] [pers. obs.], although some, such as *D. lusosso*, have been characterized as dietary generalists feeding on a range of both plant and animal materials [6]. Besides playing an important role as a major constituent of the ecologically important herbivore/detrivore guilds in African freshwaters [8], *Distichodus* is also of considerable socio-economic importance, as many species constitute a highly valued, but increasingly over-exploited, component of artisanal and commercial fisheries across the continent [9], and due to their high fecundity and herbivorous diet are increasingly being cultured in fish farms and lentic water bodies, particularly in western Africa [4].

Currently, the genus contains 25 valid species [10–12], most of which are found in the Congo River basin with species diversity decreasing with distance from that central African center of diversity (Fig. 1). Although no morphological synapomorphies have yet been identified for *Distichodus*, the genus can be distinguished from all other distichodontid genera by the combination of: an upper jaw only slightly mobile with respect to the cranium; an edentulous maxilla not tightly applied posteriorly to the premaxilla; two rows (generally) of gracile, long stalked, bicuspid teeth in each jaw; a highly mobile joint between the angulo-articular and dentary (i.e., a *Distichodus*-type lower jaw [13]); a reduced dentary portion of the mandibular sensory canal; and a completely pored lateral line [13, 14].

Morphological variation within the genus includes notable differences in overall body size, spanning two orders of magnitude and ranging from over ~ 1 m in the largest species (*D. nefasch*, *D. langi*) to ~ 5 cm in the smallest (*D. decemmaculatus*, *D. teugelsi*), lateral line scale counts (large- vs. small-scaled), the position of the mouth (terminal vs. inferior), coloration (including presence and number of dark vertical bands and spots), tooth number in the oral jaws, and fin ray counts, among others [14–16] (Fig. 1).

The genus *Distichodus* was erected in the mid-nineteenth century [17] and much of the currently recognized taxonomic diversity had been described by the early twentieth century. As is typical of the taxonomic literature prior to the mid-twentieth century, these older descriptions are highly abbreviated, usually lacking anatomical or ecological detail, and often based on examination of little or no comparative material. In one of the earliest attempts at providing a classification scheme for *Distichodus*, Boulenger [15] divided the genus in two major groups based on the number of lateral line scales. Boulenger’s classification scheme and the monophyletic status of the genus, however, were not tested until the cladistic study of Vari [13], in which the phylogenetic relationships of the Distichodontidae were investigated using comparative anatomical data. Although only five species of *Distichodus* were included in his study, Vari’s findings failed to support the hypothesis of *Distichodus* monophyly, resolving some species more closely related to a clade formed by the diminutive distichodontid genera *Nannocharax* and *Hemigrammocharax*.

Contrary to Vari’s work [13], the first molecular phylogenetic study focused on the Distichodontidae [18] found strong support for the monophyly of *Distichodus*, and while this study did not focus on the genus and sampling of *Distichodus* species was not exhaustive, it provided the first picture of *Distichodus* relationships. Despite this recent contribution to understanding of distichodontid relationships, taxonomic problems within *Distichodus* persist, and ongoing morphometric and morphological studies (Vreven, pers. comm.) indicate that considerable cryptic diversity remains unrecognized by current taxonomy [14, 16, 19]. Because the taxonomy of *Distichodus* has only been incidentally examined since the work of Boulenger [12, 13, 16, 18, 20], a comprehensive and focused phylogenetic treatment of the genus (including sampling of multiple individuals per species from a broad geographic range) is needed to test the current classification and to lay essential foundations for future investigations of this socio-economically important genus.

Therefore, to advance our understanding of the systematics and evolutionary history of *Distichodus*, in addition to providing insights into the processes generating fish diversity in freshwater environments of continental Africa, this study investigates the phylogenetic, biogeographic, and chronological framework for the diversification of the genus based on multi-locus comparative DNA sequence data. The study provides a robust phylogenetic framework for testing the adequacy of the current *Distichodus* taxonomy, informing future revisionary studies and conservation actions, as well as addressing an array of questions about the evolutionary history of the genus. Furthermore, given its pan-African distribution, knowledge on the temporal and geographic context for the diversification of *Distichodus* holds considerable promise for shedding light on the very poorly understood biogeographic history of the continent’s riverine networks.

## Results

### Sequence data summary statistics, partitioning scheme and substitution models

The concatenated alignment of eight genes consisted of 6824 sites, of which 1581 were variable and 1339 parsimony-informative. The few instances of failed DNA amplification and/or sequencing resulted in < 2% of missing data. The best partitioning scheme according to the PartitionFinder analysis comprise four partitions: 1) the entire mtDNA control region (*cr*), 2) 3rd codon positions of the protein-coding mitochondrial genes [*co1*, *cytb*, and *nd*], 3) 1st and 2nd codon positions of the nuclear genes [*enc1*, *glyt*, *myh6*, *shx3px3*] plus 2nd codon positions of the mitochondrial protein-coding genes, and 4) 3rd codon positions of nuclear genes plus 1st codon positions of mitochondrial protein-coding genes. The best-fit substitution models for these partitions were HKY + G + X, TrN + G + X, TrN + I + X, and TrNef+I + G, respectively. Models that include +X are those in which base frequencies are estimated using maximum likelihood rather than using the empirical frequency distributions.

For the BEAST2 analyses, all model parameter ESS values were greater than 200 and effective topological approximate ESS was always > 570. All best-fit codon models for individual gene trees input to ASTRAL-III were Muse and Gaut’s [21] (MG94) + M0 + F3x4 codon frequency models, with the exception of *myh6,* for which an MG94 + M3 + F3x4 model was inferred. Terminology for the number of omega (ω) classes follows Yang et al. [22].

### *Distichodus* phylogeny

**Fig. 2:**
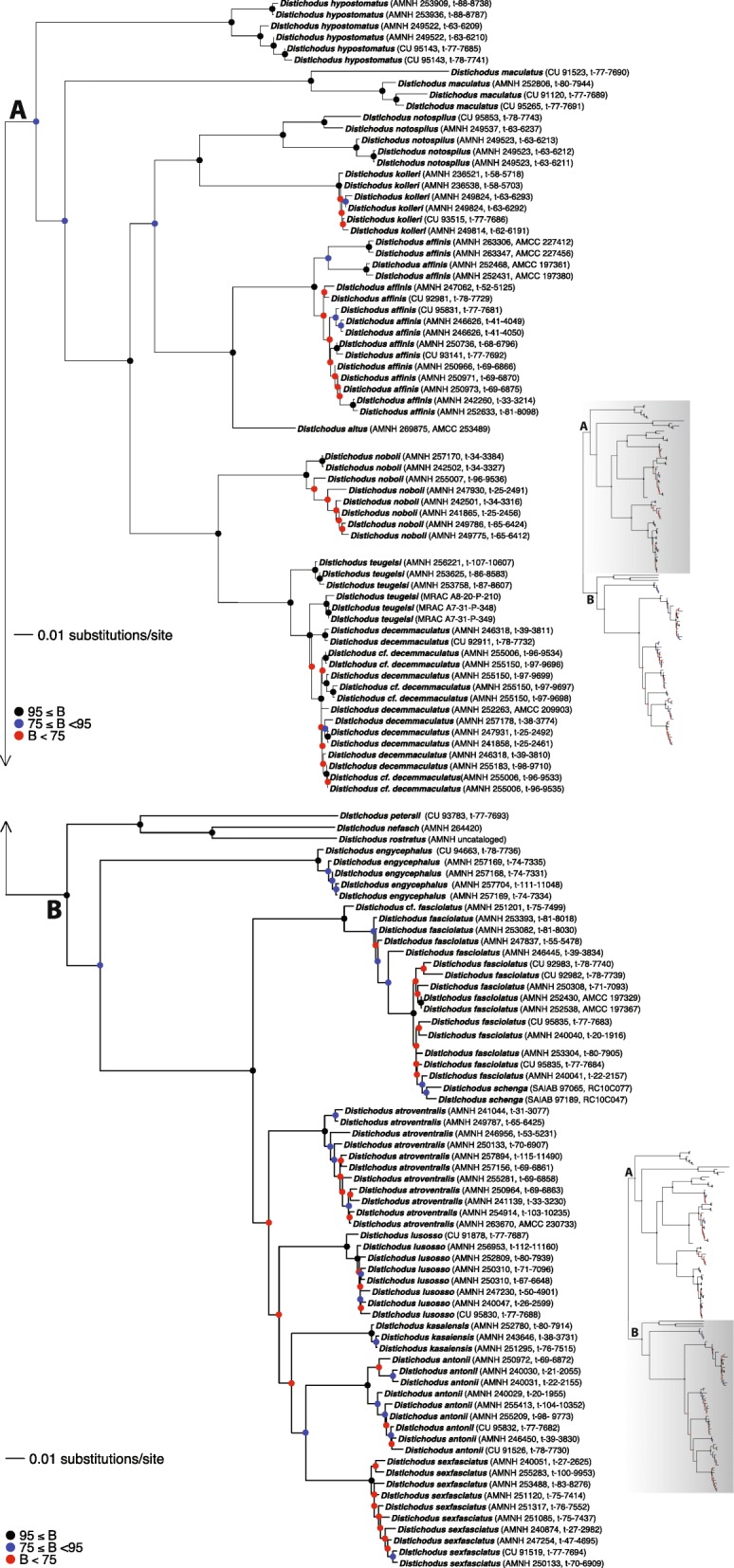
Total-evidence *Distichodus* phylogeny as inferred by likelihood in RAxML. Colored circles on nodes indicate degree of clade support as determined by bootstrap values (BS). Nodes labeled A and B represent the two main infrageneric clades. Outgroup taxon (*Paradistichodus dimiatus*) not shown

**Fig. 3:**
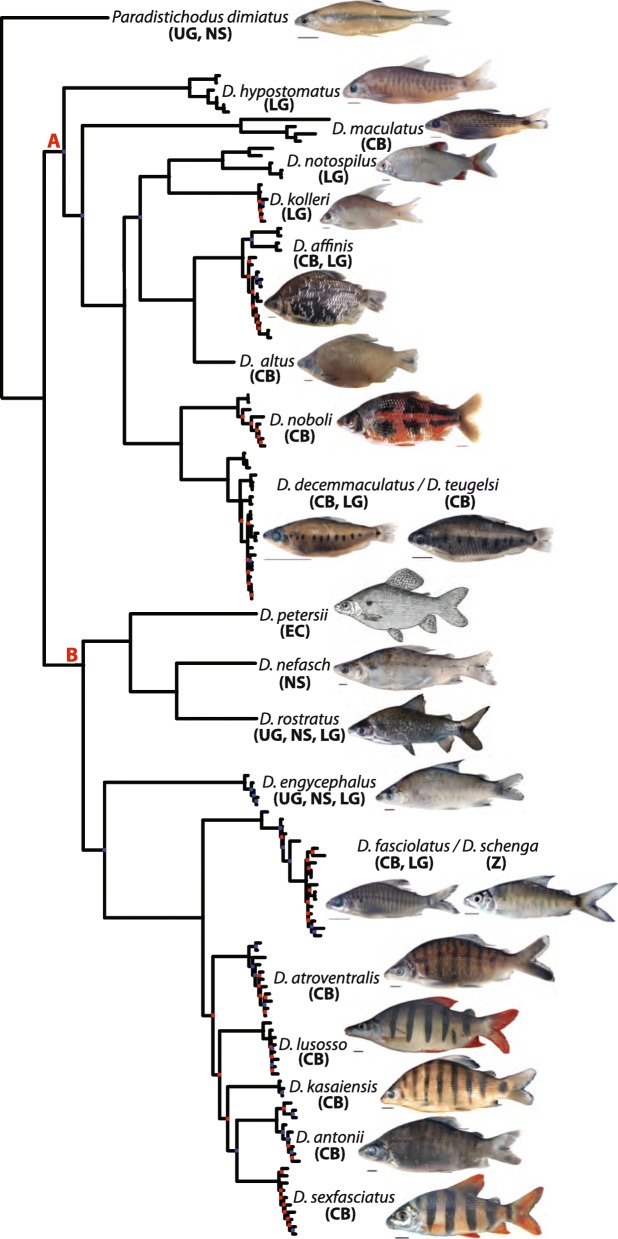
Summary tree of the RAxML *Distichodus* phylogeny highlighting interspecific relationships

The phylogeny derived from ML analysis (RAxML tree) of the concatenated alignment of all eight markers is presented in Fig. 2. A summarized version of this phylogeny, highlighting interspecific relationships, is illustrated in Fig. 3. Single-locus phylogenies (*enc1*, *glyt*, *myh6*, *sh3px3*, mtDNA) are presented in Figs. S1, S2, S3, S4 and S5, respectively. As expected, partially because of variation in substitution rates, single-locus phylogenies differed in the level of resolution and nodal support, with ncDNA markers resulting in less resolved and supported phylogenies when compared to the mtDNA locus.

**Fig. 4:**
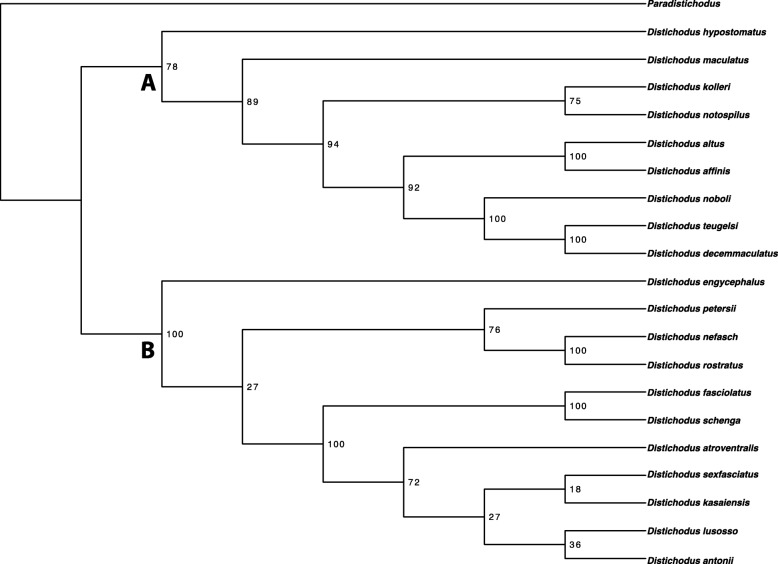
*Distichodus* species tree generated using the coalescence-based method SVDquartets

**Fig. 5:**
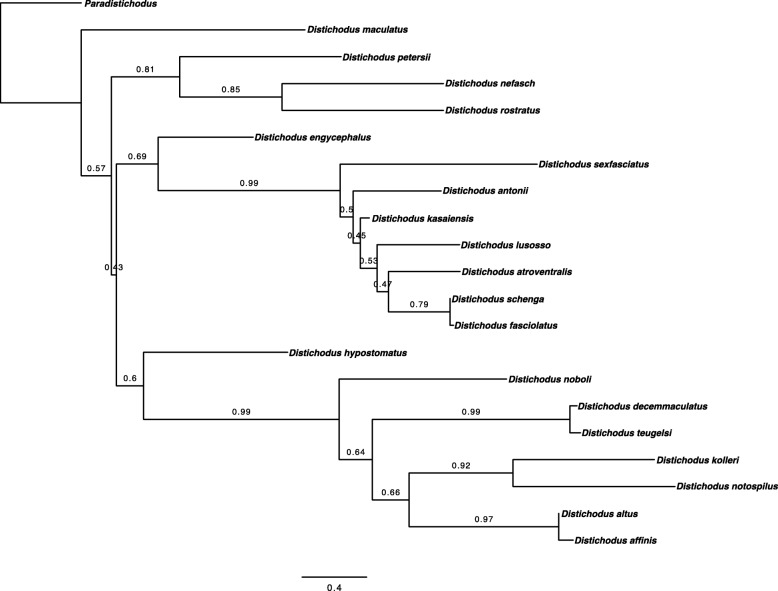
*Distichodus* species tree generated using the coalescence-based method ASTRAL-III

**Fig. 6:**
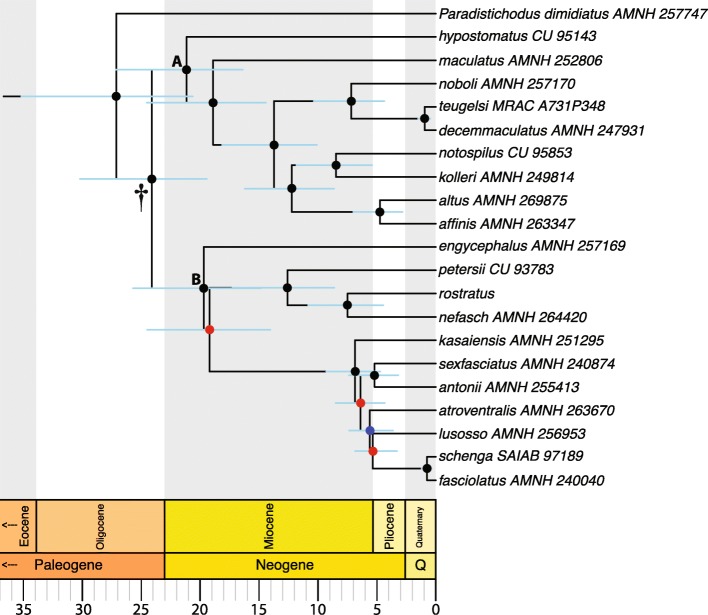
A time-scaled phylogeny of *Distichodus*. Chronogram resulting from BEAST2 analysis 8, intermediate in terms of calibration node (D, crown) and *P*_*95*_ SMB (30 Ma). Divergence-time estimates are represented by the mean ages of clades. Light red bars correspond to 95% highest posterior density (HPD) intervals of mean node ages. Calibration (fossil-based) node indicated by a dagger (†). Colored circles on nodes indicate degree of clade support as determined by posterior probabilities: black > 0.95, 0.95 ≥ blue ≥0.75, red < 0.75. Outgroup taxon (*Nannocharax ansorgii*) not shown

Species-tree analyses (SVDquartets and ASTRAL-III) results are presented in Figs. 4 and 5, respectively. BEAST2 analyses yielded very similar topologies (Figs. 6 and S6, S7, S8, S9, S10, S11, S12, S13, S14, S15, S16, S17, S18 and S19), only differing slightly in resolution within one of the two main clades discovered. The RAxML, SVDquartets, and BEAST2 phylogenies exhibit largely congruent topologies with comparable nodal support, resolving the genus into two strongly supported major clades of roughly equivalent species diversity and with the same limits and composition (clades A and B in Figs. 2, 3, 4, 6, and S6, S7, S8, S9, S10, S11, S12, S13, S14, S15, S16, S17, S18 and S19). While these three different analytical methods revealed the same general pattern of relationships in clade A, with disagreement inside clade B (notably among *D. engycephalus*, *D*. *kasaiensis*, *D. lusosso*, and *D. atroventralis*), the ASTRAL-III analysis produced a considerably different topology (Fig. 5). The source of this disagreement with the other methods is unclear.

Regardless of inference method, and conforming to expectation, nodal support was greater at deeper divergences, while weaker (BS < 75; PP < 0.75) at nodes corresponding to more recent divergences, likely reflecting intraspecific population-level structuring (when sampling multiple individuals per species). Nonetheless, for the most part, interspecific relationships are well supported, with the exception of a subclade of clade B.

### Monophyly of *Distichodus* species

Sampling of multiple individuals per species allowed testing of the monophyletic status of most morphologically diagnosed *Distichodus* species, and the resulting total evidence phylogeny (Figs. 2 and 3) strongly supports the monophyly of most of the species for which multiple individuals were sampled. However, there are two notable exceptions: the species pairs *D. teugelsi / D. decemmaculatus*, and *D. fasciolatus / D. schenga*, each of whose members were resolved as paraphyletic with respect to the other. Specifically, the phylogenetic placement of all sampled individuals of morphologically determined *D. teugelsi* renders *D. decemmaculatus* paraphyletic, and similarly, the phylogenetic placement of the two sampled individuals of *D. schenga* renders *D. fasciolatus* paraphyletic (Fig. 2). Although based on considerably fewer comparative data, the mtDNA phylogeny agreed, for the most part, with the concatenated phylogeny in the monophyly of most sampled species. Most ncDNA single-locus phylogenies, on the contrary, exhibited lower degrees of resolution and support than the total evidence and mtDNA trees, failing to support the monophyletic status of several of the species evaluated.

### Timescale of *Distichodus* diversification

The resultant chronograms from the BEAST2 analyses are presented in Figs. 6 and S6, S7, S8, S9, S10, S11, S12, S13, S14, S15, S16, S17, S18 and S19, and a summary of the results including age estimates and associated HPD intervals of select nodes in Table 1. A number of findings are apparent regardless of calibration strategy, and therefore, of absolute times of divergence. Notable among these are that *Distichodus* (crown group) originated shortly after its divergence from *Paradistichodus*, and that the two major components of the *Distichodus* radiation (clades A and B) started diversifying roughly concurrently. However, despite this initial chronological correspondence, a large subclade of clade B consisting of seven species (the MRCA of *D. kasaiensis* and *D. atroventralis* and all of its descendants) is, for the most part, of comparatively more recent origin.

**Table 1:** Results from alternative BEAST2 analyses. Estimated mean ages (in Ma) and associated 95% HPD intervals of select nodes: D + P = MRCA of *Distichodus* & *Paradistichodus*; D = MRCA of *Distichodus* species; D_ne_ + D_ro_ = MRCA of *D. nefasch* & *D. rostratus*; D_A_ = *Distichodus* subclade A; D_B_ = *Distichodus* subclade B. *P*_*95*_ SMB = 95th percentile soft maximum bound (in Ma), as a proxy for the maximum node age constraint

Analysis	Calibration node, ***P***_***95***_ SMB	D + P	D	D_**A**_	D_**B**_	D_**ne**_ + D_**ro**_
1	D + P (stem), 20	12.42 [8.71, 16.55]	11.21 [7.92, 15.14]	9.82 [6.80, 13.26]	9.14 [6.24, 12.53]	3.47 [1.98, 5.24]
2	D + P (stem), 30	15.38 [9.76, 21.86]	13.76 [8.67, 19.65]	12.01 [7.48, 17.21]	11.22 [6.94, 16.22]	4.37 [2.35, 6.81]
3	D + P (stem), 40	19.44 [11.41, 29.34]	17.47 [10.31, 26.48]	15.30 [8.87, 23.25]	14.23 [8.16, 21.66]	5.41 [2.61, 8.78]
4	D + P (crown), 20	19.03 [18.24, 20.15]	17.29 [14.75, 19.51]	15.16 [12.64, 17.63]	14.13 [11.58, 16.77]	5.38 [3.44, 7.47]
5	D + P (crown), 30	24.01 [19.43, 30.15]	21.53 [15.95, 28.01]	18.81 [13.84, 24.81]	17.60 [12.55, 23.30]	6.89 [4.04, 10.10]
6	D + P (crown), 40	28.95 [20.81, 39.58]	26.09 [17.75, 36.45]	22.89 [15.19, 32.04]	21,32 [14.00, 30.08]	8.10 [4.40, 12.38]
7	D (crown), 20	21.50 [18.54, 25.08]	19.01 [18.23, 20.07]	16.61 [14.56, 18.39]	15.54 [13.26, 17.65]	6.09 [3.98, 8.30]
8	D (crown), 30	27.12 [20.64, 35.18]	24.10 [19.44, 30.20]	21.15 [16.39, 27.14]	19.69 [14.84, 25.69]	7.49 [4.46, 10.82]
9	D (crown), 40	32.08 [22.31, 44.49]	28.60 [20.79, 38.73]	25.09 [17.90, 34.80]	23.34 [16.12, 32.41]	8.83 [4.75, 13.34]
10	D_ne_ + D_ro_ (stem), 20	41.39 [30.72, 52.63]	36.97 [28.92, 46.28]	32.44 [24.74, 41.13]	30.08 [23.66, 37.06]	11.32 [8.03, 14.69]
11	D_ne_ + D_ro_ (stem), 30	50.21 [34.81, 67.85]	44.86 [31.82, 59.25]	39.28 [27.43, 52.69]	36.53 [26.67, 48.17]	13.88 [8.93, 19.16]
12	D_ne_ + D_ro_ (stem), 40	57.55 [37.68, 80.13]	51.46 [34.88, 70.56]	45.09 [29.98, 62.81]	41.98 [28.82, 57.31]	15.90 [9.53, 22.72]
13	D_ne_ + D_ro_ (crown), 20	66.14 [43.85, 90.50]	59.09 [40.85, 79.59]	51.71 [34.59, 69.69]	48.32 [34.01, 65.26]	18.96 [18.24, 19.94]
14	D_ne_ + D_ro_ (crown), 30	78.23 [50.07, 109.73]	69.92 [45.95, 96.62]	61.16 [39.54, 85.63]	57.24 [38.17, 79.02]	22.85 [19.30, 27.43]
15	D_ne_ + D_ro_ (crown), 40	87.68 [53.18, 125.70]	78.53 [49.63, 111.69]	68.74 [43.03, 99.17]	64.28 [40.60, 91.16]	25.88 [20.21, 32.85]

Of the main variables defining calibration strategy (i.e., calibration node and *P*_*95*_ SMB), selection of calibration node appears to have the strongest effect on estimates of divergence times, with node D_ne_ + D_ro_ resulting in the oldest node age estimates (substantially older than those based on any of the other calibration nodes used), irrespective of *P*_*95*_ SMBs. However, node age estimates based on calibration node P + D did not differ considerably from those based on calibration node D, especially under equivalent *P*_*95*_ SMBs. This trend can be explained by the fact that the age difference between these nodes is relatively small, as previously mentioned. Unsurprisingly, older *P*_*95*_ SMBs resulted in older node age estimates, although perhaps not as much as anticipated.

According to the results of analysis 8 (Fig. 6), under what could be considered a “midway” calibration strategy, intermediate in terms of calibration node (D, crown) and *P*_*95*_ SMB (30 Ma), the origins of the *Distichodus* crown group date to the late Oligocene (24.1 Ma; 95% HPD = 19.44–30.20). Conforming to expectation, this estimate is older (~ 7 Ma) than the only previously published estimate, inferred in the context of a time-scaled phylogeny of the Distichodontidae (17.22 Ma; 95% HPD = 12–23) [18]. The results from analysis 8 also indicate that by the late Miocene/early Pliocene (~ 5 Ma) the bulk of species diversity in the genus was already present. Furthermore, this chronogram implies that stem lineages leading to the modern species *D. hypostomatus*, *D. maculatus*, *D. engycephalus* appeared around 21–18 Ma, while most remaining modern diversity likely originated during the late Miocene. Notably, the most recent divergences (~ 1 Ma) correspond to the seemingly paraphyletic species pairs *fasciolatus/shenga* and *teugelsi/decemmaculatus* mentioned above, an observation that supports the notion that each of these pairs may correspond to lineages at the early stages of differentiation and speciation.

### Geographic range evolution on the *Distichodus* phylogeny

**Fig. 7:**
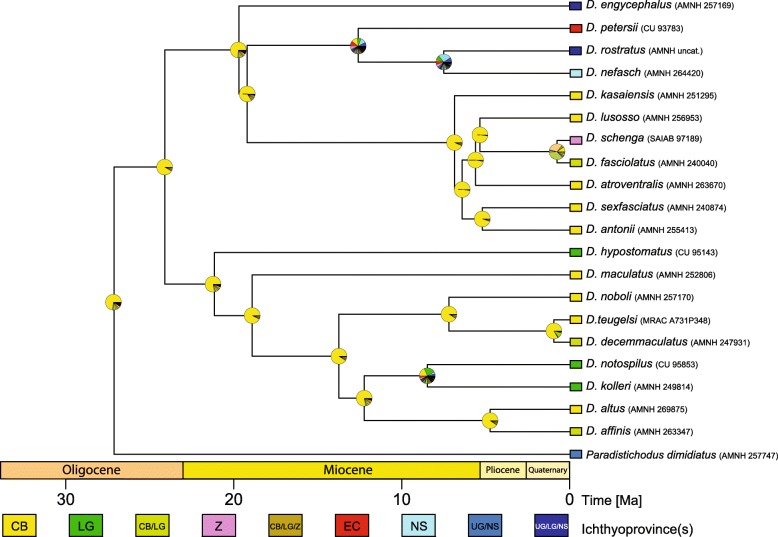
A spatiotemporal reconstruction of *Distichodus* range evolution. Based on the optimal DEC* model (M1; CB-as-source) and input chronogram resultant from BEAST2 analysis 8 (Fig. 7). Ichthyofaunal provinces color-coded and abbreviated as in Fig. 1. Probabilities of ancestral areas at each node are presented in Table S1

Model comparison using AIC and AIC weights (Table 2) indicate support for the M1 model (CB-as-source) over the M2 model (CB-as-sink), while the unconstrained (M0) model received negligible support, regardless of absolute times of divergence (input chronogram). Likewise, the pattern of range shifts out of and expansions from the Congo Basin (the ancestral area) implied by the preferred model (M1) was equivalent across analyses, irrespective of absolute node ages and despite minor topological differences between input chronograms (particularly with respect to the relative placement of *D. engycephalus*). Specifically, the M1 model inferred six range shifts for *Distichodus* out of the Congo Basin (the ancestral area) and three different range expansions from the Congo Basin to include adjacent ichthyofaunal provinces (Figs. 7, S20, and S21). Support/signal for model M1, however, appears to be stronger when based on older times of divergence (Table 2).

**Table 2:** Results from DEC* analysis of geographic range evolution on the *Distichodus* phylogeny. Results are presented for each of the three analyses based on different BEAST2 input chronograms (derived from analyses 5, 8, and 14). Comparison of alternative models (biogeographic hypotheses) and their support as assessed via Akaike weights. M0 (unconstrained, dispersal to and from the Congo Basin); M1 (allowing only dispersal out of the Congo Basin); M2 (allowing only dispersal into the Congo Basin); dispersal (d); extinction (e); number of parameters (k); Akaike information criterion (AIC); Akaike Weights (AW)

Input chronogram	Hypotheses (constraints)	lnL	Parameter estimates			AIC analysis	
			k	d	e	AIC	AW
Analysis 5	M0	−52.01724	2	0.01911367	0.10630582	108.03450	0.08844
	**M1**	**−49.86777**	**2**	**0.02413022**	**0.10000542**	**103.73550**	**0.75880**
	M2	−51.47061	2	0.01772728	0.02239203	106.94120	0.15277
Analysis 8	M0	−52.19156	2	0.01734394	0.0977318	108.38310	0.08778
	**M1**	**−49.87853**	**2**	**0.02169815**	**0.09103859**	**103.75710**	**0.88703**
	M2	−53.44013	2	0.01639595	0.02140229	110.88030	0.02519
Analysis 14	M0	−52.17641	2	0.006007077	0.033905864	108.35280	0.08558
	**M1**	**−49.83422**	**2**	**0.007466523**	**0.03069**	**103.66840**	**0.89043**
	M2	−53.44833	2	0.005663481	0.007312191	110.89670	0.02399

## Discussion

### *Distichodus* relationships and taxonomic implications

Here we present the first comprehensive phylogenetic, temporal, and biogeographic framework for examination of the current taxonomy and evolutionary history of *Distichodus* and for future evolutionary studies of the genus. Regardless of analytical method (except for ASTRAL-III, but see below), our results, based on a dataset with considerably more inclusive taxon, character, and geographic sampling for *Distichodus* than previous works, strongly support the existence of two roughly equal-sized, and reciprocally monophyletic lineages within the genus, while corroborating the monophyletic status of most currently recognized species. RAxML, SVDquartets, and BEAST2 topologies are largely congruent, with some swapping of taxa inside the two-clade *Distichodus* structure that is also supported by morphology [13]. Only the ASTRAL-III analysis did not conform to this general picture of *Distichodus* relationships, but there are two confounding issues then at play. First, ASTRAL-III may be sensitive to gene tree estimation error. The codon model approach to gene tree inference used here should, in principle, be the most accurate method for gene tree inference (of those currently available reversible Markov models), given its hierarchical modeling structure [23]. However, it is still not immune to the requirements of large amounts of data [24]. Some close-to-zero-length branches in the individual gene trees may either be (1) a true artifact of ILS, or (2) a consequence of insufficient data (in terms of gene length, or in terms of evolutionary rate distribution). However, distinguishing differences between real ILS and insufficient data is not possible from the current analysis. We therefore focused our attention and present our conclusions based on the results with the largest overall congruence.

Conforming to expectation, nodal support tends to be higher at more basal nodes (deeper divergences), whereas more recent divergences are, on average, less strongly supported. Low nodal support and instances of conflict between analytical methods of phylogenetic inference are particularly evident for the clade consisting of the predominantly large-bodied species (*D. atroventralis, D. lusosso, D. kasaiensis, D. antonii,* and *D. sexfasciatus*), suggesting that additional data will be necessary to resolve interspecific relationships for this particular section of the tree. Disagreement between methods in this part of the *Distichodus* tree is an aspect worth revisiting in the future with genome-wide NGS-generated data, as larger amounts of DNA sequence data might be capable of better resolving and supporting these divergences. Besides the obvious reasons for wanting to unambiguously resolve this part of the *Distichodus* tree, such an endeavor is of special interest because the highly disparate trophic-related morphologies displayed by members of this clade are undoubtedly an interesting character system from both evolutionary ecological and functional morphological perspectives.

Despite some of the disagreements between inference methods, our results offer a general working hypothesis of *Distichodus* relationships and, with few exceptions, are consistent with the current species-level morphology-based taxonomy of the group. Instances of questionable species monophyly and therefore in conflict with the current classification are discussed below.

#### Paraphyly of *D. teugelsi* with respect to *D. decemmaculatus*

Problems with the species recognition of the two dwarf species, *D. decemmaculatus* and *D. teugelsi,* have been noted by Verheyen et al. [19], and are confirmed here. Species identification has previously been based on the presence (*decemmaculatus*) or absence (*teugelsi*) of a series of dark spots or bars along the flanks, and 20 (*decemmaculatus*) versus 16 (*teugelsi*) scales around the caudal peduncle [11]. While our study finds strong support for a *teugelsi*/*decemmaculatus* clade, samples tentatively identified as *D. teugelsi* from the Kwilu River in the Kasai basin (with 16–17 scales around the caudal peduncle and variously marked spots or bars along the flanks), form a well-supported sister clade to the remaining samples. While samples of *D. teugelsi* from the type locality, the Lefini River, a right bank tributary of the Congo River upstream of Pool Malebo (lacking spots or bars on the flanks and with 16 scales around the caudal peduncle), form a clade sister to the *D. decemmaculatus* samples, all tentatively identified here as *D. decemmaculatus* or *D.* cf. *decemmaculatus*. Among these we record caudal peduncle scales counts ranging from 18 to 20, and flank pigmentation ranging from virtually absent to clearly marked and strongly spotted. While no taxonomic solution is proposed here, based on the molecular analysis presented and the observation of high variability in both pigmentation and scale counts in geographically disparate samples of both “species”, further study of the *teugelsi/decemmaculatus* clade, including representatives of populations across the range of each putative taxon, is needed. We note further that, as for the *fasciolatus*/*shenga* species pair discussed below, our estimation of the divergence time for the *teugelsi*/*decemmaculatus* pair (based on analysis 8; Fig. 6) is among the most recent (~ 1 Ma). The fact that this is a very recent divergence, might explain the resulting paraphyletic pattern. It is well known that genetic variation shared between closely related species can be due to retention of ancestral genetic polymorphisms resulting from incomplete lineage sorting (ILS) [25], a process that can confound phylogenetic inference and hinder robust tests of monophyly in recently diverged species pairs. Whereas mtDNA introgressive hybridization has been also recognized as one cause of misleading inferences of paraphyly, the overall congruence between the nc- and mtDNA signal involving the *teugelsi/decemmaculatus* pair supports ILS instead of introgression as a probable explanation for the observed pattern of paraphyly [26].

#### Paraphyly of *D. fasciolatus* with respect to *D. schenga*

Representatives of the widespread species *D. fasciolatus* are rendered paraphyletic by the placement of the two sampled individuals of the southern African species, *D. schenga,* a middle and lower Zambezi endemic, which are placed well nested within a strongly supported *D. fasciolatus* clade (Fig. 2). *Distichodus schenga* (type locality Tete, Zambesi River) was described by Peters in 1852 and *D. fasciolatus* by Boulenger in 1898 (type localities in the lower Congo River region), and the descriptions of both are minimal, not allowing for morphological species discrimination. Possibly because of this, Boulenger [15] did not include *D. schenga* in his key to *Distichodus*, and by implication did not recognize it as distinct from *D. fasciolatus*. Our molecular data clearly suggest that the synonomy of *D. fasciolatus* with *D. schenga* is in order, however ongoing morphometric and morphological study of the entire “*fasciolatus*-complex” is currently underway (Vreven, pers. comm.), and pending the results of that study we defer proposing a formal taxonomic synonomy based solely on our molecular data and minimal sampling of putative *D. schenga* from across the Zambezi basin.

We do note however, that the phylogenetic and chronological pattern revealed by our study (Figs. 2, 4, and 6), coupled with the allopatric distribution of these two taxa, suggest that populations currently recognized as *D. schenga* could have diverged from a lineage/population of *D. fasciolatus* that colonized the Upper Zambezi headwaters from the Kasai during the Pleistocene, when the two river systems shared a past connection [27–29]. This chronological and geographic dispersal scenario out of the Congo Basin is consistent with our estimated divergence time for this species pair (~ 1 Ma) (Fig. 6) and the inferred range shift involving *D. schenga* (Fig. 7), and has been hypothesized for various other fish taxa across the headwaters of the Congo-Zambezi watersheds [30–32].

In any case, a phylogenetic pattern of short, poorly supported branches is an indicator of recent species divergence that precluded mtDNA lineages from sorting to reciprocal monophyly [33]. Therefore, as for the *teugelsi*/*decemmaculatus* pair, we cannot rule out the possibility that the inferred paraphyly of *D. fasciolatus* with respect to *D. schenga* is an artifact of ILS issues. However, signal discordance between mtDNA and nuclear markers involving the *fasciolatus/schenga* pair strongly suggest that post-divergence introgressive hybridization could also explain the inferred paraphyly of *D. fasciolatus* with respect to *D. schenga*.

### A spatiotemporal framework for *Distichodus* diversification

In the context of their time-scaled phylogeny of the suborder Citharinoidei, Arroyave et al. [18] were among the first authors to estimate an age for the origin of *Distichodus* and the timing of diversification within the genus. Their chronogram suggested that the *Distichodus* crown group appeared in the Miocene (~ 17 Ma), but that most of the species diversity likely originated during the past 5 Ma. These inferences were based on a molecular clock calibrated using ~ 7.5 Ma *Distichodus* fossilized dentition [34], which at the time was the oldest known fossil assignable to the genus. The recent discovery of a considerably older (18–19 Ma) *Distichodus* fossil [35], however, prompted our reexamination of the timescale of *Distichodus* diversification in the context of a larger dataset, both in terms of molecular markers (8 vs. 7 loci) and taxon sampling (20 vs. 16 spp.). This older fossil, however, presented us with the challenge of accurately assigning it to a node for the purpose of calibrating the molecular clock and estimating absolute times of divergence in the phylogeny of *Distichodus*.

Whereas the approach devised herein to address the uncertainties associated with the fossil-based calibration of the molecular clock resulted in multiple alternative chronograms, from our knowledge of the study subject we believe that some of these alternative calibration scenarios might be either overly conservative (e.g., D + P stem) or too liberal (e.g., D_ne_ + D_ro_ crown), therefore possibly resulting in under- or overestimation of node ages, respectively. Nonetheless, because we have no means to empirically falsify any of these alternative calibration scenarios, we consider it important and valuable to offer the reader the possibility of choosing among alternative scenarios (including those we think too extreme) based on their own knowledge of the study subject and their personal beliefs regarding best practices for justifying fossil calibrations [36].

For the most part, our findings imply a temporal framework for the diversification of *Distichodus* older than previously reported [18], irrespective of calibration strategy. Only analyses 1 and 2, based on calibration node D + P (stem), resulted in younger divergence time estimates (Table 1). While at present we feel more comfortable grounding our discussion of the temporal and geographic context for the diversification of *Distichodus* in the results from analysis 8 (intermediate in terms of calibration node [D, crown] and *P*_*95*_ SMB [30 Ma]), we acknowledge that, should this calibration fossil be eventually confirmed as *D. nefasch, D. rostratus*, or their MRCA (a possibility due to fossil tooth shape, size, and geographic distribution), a reinterpretation of the biogeographic history will be necessary to reconcile the inferred patterns of geographic range evolution with a chronological framework more than twice as old as the one discussed below (Fig. S21).

Despite the high ichthyofaunal diversity of Afrotropical continental waters, few studies have investigated the chronological and biogeographic context for the diversification of African freshwater fish clades, among which only a handful have focused on Pan-African riverine genus-level radiations, namely *Hydrocynus* [30], *Mastacembelus* [37, 38] and the species-rich *Synodontis* [32, 39]. Notably, a Miocene diversification for *Distichodus*, as implied by the chronogram resulting from analysis 8 (Fig. 6), broadly concurs with previous findings for both *Mastacembelus* and *Synodontis* [32, 38, 39]. Similarly, a concurrence of Miocene diversification among various lineages of fishes, frogs, and crabs has been pointed out by Daniels et al. [40], who suggest this likely reflects a shared response to mesic climatic shifts resulting in marked allopatric differentiation among each of these freshwater lineages during the mid- to late Miocene. While to our knowledge there are no empirical studies proving a causal relationship between particular paleohydrological events and diversification patterns in African freshwater fishes, some authors have suggested that Miocene tectonic and climatic upheaval may have influenced or even triggered diversification [31, 38]. The Miocene geological epoch was the setting for widespread epeirogenic uplift in Africa and global climate change that profoundly contributed to shaping the modern African hydrological landscape [41–43], which in turn, it is believed, promoted diversification in freshwater fishes as a consequence of river discharge shifts (due to climate change) and drainage disruption and modification (due to rifting) [44]. Our findings about the timing of *Distichodus* diversification add to instances of Miocene continent-wide freshwater radiations, and therefore to a growing body of evidence in support for a “hydrogeological” hypothesis, that paleohydrological and paleoclimatic changes promote landscape evolution which in turn promotes cladogenesis in freshwater organisms [45, 46]. Further research, however, ideally in a multi-taxon comparative framework, is certainly needed to robustly test hypotheses of concerted responses to paleogeologic and paleoclimatic scenarios.

Analysis of geographic range evolution on the phylogeny of *Distichodus* favored a biogeographic model in which the Congo Basin (CB) is the center of origin (ancestral area) and source of the geographic diversity of the genus, irrespective of absolute times of divergence. In particular, the biogeographic reconstruction based on the chronogram resulting from analysis 8 (Fig. 7), implies that most cladogenetic events occurred in lineages still confined to the CB throughout most of the Miocene, but also multiple lineage range shifts out of and expansions from the CB into adjacent ichthyofaunal provinces at different times during the evolutionary history of the group. Only in the late Miocene (~ 9–7 Ma) are the first recorded instances of range shifts out of the CB and of cladogenesis occurring in other ichthyofaunal provinces, namely the Nilo-Sudan (NS) and Lower Guinea (LG). The remaining instances of range shifts and expansions are more recent, dating back to the Pliocene. While most ichthyofaunal provinces appear to have been colonized only once (or twice in the case of UG), our results indicate that LG was independently colonized by five different lineages, mostly during the Pliocene.

Our reconstruction of the biogeographic history of *Distichodus* suggests a central role of the CB in the distribution of the continent’s freshwater ichthyofauna during the late Cenozoic, offering support to the hypothesis that the CB is the source of the ichthyofauna of less diverse river basins throughout continental Africa [44]. While a CB origin has also been postulated for the African tigerfish *Hydrocynus* [30], other continent-wide African freshwater fish genera such as *Synodontis* [32] and *Mastacembelus* [38] do not conform to this pattern and suggest repeated independent colonization into the CB. Considering the vast geographic area under study, and that complex evolutionary histories of dispersal and vicariance are likely to exist among the different fish lineages, these conflicting biogeographic histories certainly suggest a complex scenario for the assemblage of the continent’s ichthyofaunal communities.

## Conclusions

The spatiotemporal framework for the diversification of African freshwater fish genus *Distichodus* presented herein provides a significant advance in our knowledge of the evolutionary history of this ecologically and socio-economically important group of fishes. With few exceptions, the resulting phylogeny is consistent with the current species-level taxonomy of the group, offering a working hypothesis of *Distichodus* relationships that will serve as phylogenetic framework for future evolutionary studies involving phenotypic and genomic systems. The few instances of species paraphyly (*D. teugelsi, D. fasciolatus*) revealed in our favored phylogeny are likely a consequence of introgression and/or incomplete lineage sorting due to recent speciation. Therefore, we refrain from making taxonomic/nomenclatural changes pending further morphological assessment based on a larger sample of comparative material. While analysis of geographic range evolution favored a biogeographic scenario in which the Congo Basin is the source of geographic diversity of the genus, this finding is both in agreement and conflict with previous studies of other continent-wide African freshwater fish genera, suggesting a complex scenario for the assemblage of Africa’s continental ichthyofaunal communities.

## Methods

### Taxon sampling

Ingroup sampling consisted of 133 specimens representing 20 of the 25 valid *Distichodus* species, thereby encompassing 80% of *Distichodus* currently recognized diversity (Table 3). *Distichodus brevipinnis*, *D. langi*, *D. mossambicus*, *D. rufigiensis*, and the newly described *D. ingae* [12], were not included in analyses due to unavailability of tissues. With the exception of *D. altus*, *D. nefasch*, *D. rostratus*, and *D. petersii*, for which only a single tissue sample was available, multiple individuals per species were sequenced to sample as large a portion of each species’ range as possible (Table 3). In addition to increasing geographic coverage, inclusion of multiple individuals per species allowed for testing the monophyletic status—and therefore species limits under the phylogenetic species concept [47, 48]––of nominal species from which more than one individual was available for sequencing. Sampling of multiple individuals per species, however, was not aimed at making inferences about tokogenetic (intraspecific) relationships and/or phylogeographic patterns. *Paradistichodus dimidiatus* was included as outgroup based on the findings from a relatively recent molecular phylogenetic study that investigated relationships of the Distichodontidae [18], which resolved the monotypic genus *Paradistichodus* as the sister group of *Distichodus*. Similarly, *Nannocharax ansorgii* was included as additional and outermost outgroup for molecular dating and inference of geographic range evolution analyses.

**Table 3:** Taxa, voucher specimens (catalog and tissue numbers), and GenBank accession numbers for the gene sequences included in the analyses. Institutional abbreviations: AMCC (Ambrose Monell CryoCollection, AMNH), AMNH (American Museum of Natural History), CU (Cornell University Museum of Vertebrates), SAIAB (South African Institute for Aquatic Biodiversity), MRAC (Royal Museum for Central Africa)

Taxon	Catalog #	Voucher/Tissue #	Country	Ichthyofaunal province, drainage	GenBank Accession Number							
					***co1***	***cr***	***cytb***	***enc1***	***glyt***	***myh6***	***nd2***	***sh3px3***
*Nannocharax ansorgii*	AMNH 257013	t-111-11,018	Guinea	UG	KF541815	n/a	KF541951	KF542063	KF542157	KF542230	KF542408	KF542486
*Paradistichodus dimidiatus*	AMNH 257747	t-110-10,981	Guinea	UG	KF541830	n/a	KF541914	KF542040	KF542146	KF542266	KF542390	KF542497
*Distichodus affinis*	AMNH 263347	AMCC 227456	Democratic Republic of Congo	CB, Congo R., Boma.	MT300571	MT301534	MT300757	MT300808	MT300942	MT301157	MT301230	MT301317
*Distichodus affinis*	AMNH 263306	AMCC 227412	Democratic Republic of Congo	CB, Congo R., Boma.	MT300572	MT301535	MT300758	MT300801	MT300940	MT301158	MT301231	MT301316
*Distichodus affinis*	AMNH 252431	AMCC 197380	Democratic Republic of Congo	CB, Kwilu R.	MT300569	MT301548	MT300759	MT300798	MT300932	MT301152	MT301228	MT301349
*Distichodus affinis*	AMNH 246626	t-41-4050	Democratic Republic of Congo	CB, Congo R., Luozi.	MT300577	MT301542	MT300767	MT300804	MT300936	MT301160	MT301234	MT301356
*Distichodus affinis*	AMNH 250971	t-69-6870	Democratic Republic of Congo	CB, N’Sele R.	MT300578	MT301543	MT300768	MT300805	MT300937	MT301161	MT301237	MT301360
*Distichodus affinis*	AMNH 250736	t-68-6796	Democratic Republic of Congo	CB, N’Sele R.	MT300581	MT301536	MT300769	MT300806	MT300948	MT301162	MT301242	MT301357
*Distichodus affinis*	AMNH 250973	t-69-6875	Democratic Republic of Congo	CB, N’Sele R.	MT300582	MT301544	MT300770	MT300795	MT300931	MT301163	MT301238	MT301358
*Distichodus affinis*	AMNH 250966	t-69-6866	Democratic Republic of Congo	CB, N’Sele R.	MT300583	MT301545	MT300771	MT300807	MT300944	MT301164	MT301239	MT301359
*Distichodus affinis*	CU 92981	t-78-7729	Gabon	LG, Lekoli R.	MT300584	MT301547	MT300772	MT300796	MT300945	MT301165	MT301240	MT301361
*Distichodus affinis*	AMNH 252468	AMCC 197361	Democratic Republic of Congo	CB, Kwilu R.	MT300570	MT301549	MT300760	MT300799	MT300935	MT301153	MT301229	MT301350
*Distichodus affinis*	AMNH 252633	t-81-8098	Democratic Republic of Congo	CB, Lulua R.	MT300574	MT301538	MT300762	MT300800	MT300933	MT301159	MT301235	MT301351
*Distichodus affinis*	CU 95831	t-77-7681	Gabon	LG, Lekoli R.	MT300579	MT301540	MT300763	MT300797	MT300938	MT301154	MT301232	MT301352
*Distichodus affinis*	AMNH 242260	t-33-3214	Democratic Republic of Congo	CB, Congo R., Luozi.	MT300575	MT301539	MT300764	MT300794	MT300934	MT301155	MT301236	MT301353
*Distichodus affinis*	AMNH 247062	t-52-5125	Democratic Republic of Congo	CB, Congo R., Luozi.	MT300580	MT301546	MT300765	MT300802	MT300947	MT301075	MT301241	MT301354
*Distichodus affinis*	AMNH 246626	t-41-4049	Democratic Republic of Congo	CB, Congo R., Luozi.	MT300576	MT301541	MT300766	MT300803	MT300943	MT301156	MT301233	MT301355
*Distichodus affinis*	CU 93141	t-77-7692	Gabon	LG, Lekoli R.	MT300573	MT301537	MT300761	MT300809	MT300946	MT301166	MT301243	MT301345
*Distichodus altus*	AMNH 269875	AMCC 253489	Democratic Republic of Congo	CB, Lake MaiNdombe	MT300568	MT301533	MT300756	MT300824	MT300941	MT301171	MT301227	n/a
*Distichodus antonii*	AMNH 255413	t-104-10,352	Democratic Republic of Congo	CB, Congo R., Ngombe.	MT300643	MT301465	MT300693	MT300867	MT300998	MT301058	MT301275	MT301395
*Distichodus antonii*	CU 95832	t-77-7682	Democratic Republic of Congo	CB, Congo R., Wanie-Rukula.	MT300641	MT301466	MT300689	MT300870	MT301028	MT301055	MT301277	MT301396
*Distichodus antonii*	AMNH 240031	t-22-2155	Republic of Congo	CB, Congo R., Bela.	MT300639	MT301469	MT300687	MT300884	MT301029	MT301052	MT301271	MT301397
*Distichodus antonii*	AMNH 246450	t-39-3830	Democratic Republic of Congo	CB, N’Djili R.	MT300647	MT301467	MT300690	MT300878	MT301006	MT301056	MT301278	MT301398
*Distichodus antonii*	AMNH 240030	t-21-2055	Republic of Congo	CB, Congo R., Foulakari.	MT300642	MT301470	MT300691	MT300879	MT300997	MT301053	MT301272	MT301399
*Distichodus antonii*	AMNH 250972	t-69-6872	Democratic Republic of Congo	CB, N’Sele R.	MT300640	MT301471	MT300688	MT300885	MT301001	MT301057	MT301273	MT301400
*Distichodus antonii*	AMNH 255209	t-98-9773	Democratic Republic of Congo	CB, Congo R., Nkana.	MT300644	MT301468	MT300694	MT300868	MT300999	MT301059	MT301279	MT301401
*Distichodus antonii*	AMNH 240029	t-20-1955	Democratic Republic of Congo	CB, Pool Malebo.	MT300645	MT301463	MT300692	MT300880	MT301000	MT301054	MT301276	MT301402
*Distichodus antonii*	CU 91526	t-78-7730	Central African Republic	CB, Oubangui R.	MT300646	MT301464	MT300695	MT300869	MT301005	MT301060	MT301274	MT301403
*Distichodus atroventralis*	AMNH 263670	AMCC 230733	Democratic Republic of Congo	CB, Congo R., Boma.	MT300625	MT301485	MT300717	MT300894	n/a	MT301095	n/a	MT301414
*Distichodus atroventralis*	AMNH 246956	t-53-5231	Democratic Republic of Congo	CB, Congo R., Luozi.	MT300615	MT301477	MT300708	MT300886	MT301018	MT301086	MT301283	MT301404
*Distichodus atroventralis*	AMNH 249787	t-65-6425	Democratic Republic of Congo	CB, Lomako R.	MT300617	MT301475	MT300709	MT300887	MT301023	MT301087	MT301281	MT301405
*Distichodus atroventralis*	AMNH 250964	t-69-6863	Democratic Republic of Congo	CB, N’Sele R.	MT300621	MT301483	MT300710	MT300888	MT301015	MT301088	MT301286	MT301406
*Distichodus atroventralis*	AMNH 250133	t-70-6907	Democratic Republic of Congo	CB, Congo R., Luozi.	MT300618	MT301478	MT300711	MT300881	MT301024	MT301089	MT301287	MT301407
*Distichodus atroventralis*	AMNH 255281	t-69-6858	Democratic Republic of Congo	CB, N’Sele R.	MT300622	MT301481	MT300712	MT300889	MT301019	MT301090	MT301280	MT301408
*Distichodus atroventralis*	AMNH 257156	t-69-6861	Democratic Republic of Congo	CB, N’Sele R.	MT300619	MT301479	MT300713	MT300890	MT301020	MT301091	MT301282	MT301409
*Distichodus atroventralis*	AMNH 241044	t-31-3077	Democratic Republic of Congo	CB, Congo R., Bulu.	MT300616	MT301476	MT300718	MT300891	MT301021	MT301092	MT301284	MT301410
*Distichodus atroventralis*	AMNH 241139	t-33-3230	Democratic Republic of Congo	CB, Congo R., Luozi.	MT300623	MT301482	MT300714	MT300882	MT301016	MT301093	MT301285	MT301411
*Distichodus atroventralis*	AMNH 254914	t-103-10,235	Democratic Republic of Congo	CB, Mai-Ndombe R.	MT300624	MT301484	MT300715	MT300892	MT301017	MT301062	MT301288	MT301412
*Distichodus atroventralis*	AMNH 257894	t-115-11,490	Democratic Republic of Congo	CB, Congo R., Ngombe.	MT300620	MT301480	MT300716	MT300893	MT301025	MT301094	MT301289	MT301413
*Distichodus decemmaculatus*	AMNH 247931	t-25-2492	Democratic Republic of Congo	CB, Luilaka R., Monkoto.	MT300549	MT301512	MT300748	MT300844	MT300956	MT301126	MT301216	MT301333
*Distichodus decemmaculatus*	AMNH 255150	t-97-9699	Democratic Republic of Congo	CB, Mai-Ndombe R.	MT300561	MT301519	MT300742	MT300840	MT300953	MT301132	MT301220	MT301338
*Distichodus decemmaculatus*	AMNH 252263	AMCC 209903	Democratic Republic of Congo	CB, Luilaka R., Bosombangwa.	MT300556	MT301514	MT300740	MT300835	MT300952	MT301127	MT301219	MT301348
*Distichodus decemmaculatus*	CU 92911	t-78-7732	Gabon	LG, Lekoli R.	MT300544	MT301520	MT300738	MT300838	MT300965	MT301128	MT301209	MT301334
*Distichodus decemmaculatus*	AMNH 241858	t-25-2461	Democratic Republic of Congo	CB, Lofombo R.	MT300550	MT301513	MT300749	MT300845	MT300957	MT301129	MT301217	MT301335
*Distichodus decemmaculatus*	AMNH 246318	t-39-3811	Democratic Republic of Congo	CB, Lengoue R., Louesso.	MT300545	MT301521	MT300739	MT300839	MT300939	MT301130	MT301210	MT301336
*Distichodus* cf. *decemmaculatus*	AMNH 255150	t-97-9698	Democratic Republic of Congo	CB, Mai-Ndombe R.	MT300559	MT301517	MT300741	MT300836	MT300954	MT301131	MT301214	MT301337
*Distichodus decemmaculatus*	AMNH 255183	t-98-9710	Democratic Republic of Congo	CB, Mai-Ndombe R.	MT300551	MT301510	MT300750	MT300837	MT300966	MT301133	MT301223	MT301339
*Distichodus decemmaculatus*	AMNH 257178	t-38-3774	Democratic Republic of Congo	CB, Yenge R., Boyenga.	MT300552	MT301507	MT300744	MT300846	MT300967	MT301123	MT301218	MT301340
*Distichodus decemmaculatus*	AMNH 246318	t-39-3810	Republic of Congo	CB, Lengoue R., Louesso.	MT300553	MT301508	MT300753	MT300847	MT300955	MT301134	MT301226	MT301341
*Distichodus* cf. *decemmaculatus*	AMNH 255150	t-97-9696	Democratic Republic of Congo	CB, Mai-Ndombe R.	MT300557	MT301515	MT300754	MT300849	MT300960	MT301124	MT301221	MT301330
*Distichodus* cf. *decemmaculatus*	AMNH 255006	t-96-9533	Democratic Republic of Congo	CB, Mai-Ndombe R.	MT300554	MT301509	MT300751	MT300841	MT300961	MT301135	MT301224	MT301331
*Distichodus* cf. *decemmaculatus*	AMNH 255006	t-96-9534	Democratic Republic of Congo	CB, Mai-Ndombe R.	MT300558	MT301516	MT300755	MT300842	MT300962	MT301136	MT301222	MT301332
*Distichodus* cf. *decemmaculatus*	AMNH 255150	t-97-9697	Democratic Republic of Congo	CB, Mai-Ndombe R.	MT300560	MT301518	MT300743	MT300834	MT300949	MT301125	MT301215	MT301343
*Distichodus* cf. *decemmaculatus*	AMNH 255006	t-96-9535	Democratic Republic of Congo	CB, Mai-Ndombe R.	MT300555	MT301511	MT300752	MT300843	MT300964	MT301137	MT301225	MT301344
*Distichodus engycephalus*	AMNH 257169	t-74-7334	Guinea	NS, Niger R, Diaragbela.	MT300591	MT301443	MT300659	MT300784	MT300984	MT301112	MT301246	MT301372
*Distichodus engycephalus*	AMNH 257169	t-74-7335	Guinea	NS, Niger R, Diaragbela.	MT300592	MT301445	MT300660	MT300785	MT300985	MT301051	MT301248	MT301373
*Distichodus engycephalus*	AMNH 257168	t-74-7331	Guinea	UG, Dion R.	MT300593	MT301444	MT300661	MT300786	MT300986	MT301113	MT301249	MT301374
*Distichodus engycephalus*	AMNH 257704	t-111-11,048	Guinea	UG	MT300594	MT301446	MT300662	MT300787	MT300987	MT301114	MT301247	MT301375
*Distichodus engycephalus*	CU 94663	t-78-7736	Ethiopia	NS, Alwero R.	MT300590	MT301442	n/a	n/a	n/a	MT301061	n/a	MT301415
*Distichodus fasciolatus*	AMNH 240040	t-20-1916	Democratic Republic of Congo	CB, Pool Malebo, Kintele, RC	MT300604	MT301451	MT300668	MT300876	MT301034	MT301097	MT301296	MT301381
*Distichodus fasciolatus*	AMNH 246445	t-39-3834	Democratic Republic of Congo	CB, N’Djili R.	MT300603	MT301458	MT300666	MT300875	MT301014	MT301104	MT301291	MT301291
*Distichodus fasciolatus*	AMNH 250308	t-71-7093	Democratic Republic of Congo	CB, Congo R., Luozi.	MT300607	n/a	MT300675	MT300866	MT301027	MT301105	MT301298	MT301388
*Distichodus fasciolatus*	AMNH 247837	t-55-5478	Democratic Republic of Congo	CB, Lulua R.	MT300600	MT301461	MT300665	MT300898	MT301010	MT301106	MT301304	MT301389
*Distichodus fasciolatus*	AMNH 251201	t-75-7499	Democratic Republic of Congo	CB, Lulua R., DRC	MT300601	MT301462	MT300707	MT300871	MT301011	MT301107	MT301292	MT301390
*Distichodus fasciolatus*	AMNH 253393	t-81-8018	Democratic Republic of Congo	CB, Kasai R., DRC	MT300602	MT301460	MT300663	MT300877	MT301012	MT301108	MT301293	MT301391
*Distichodus fasciolatus*	AMNH 253304	t-80-7905	Democratic Republic of Congo	CB, Lulua R.	MT300605	MT301450	MT300671	MT300895	MT301022	MT301098	MT301306	MT301382
*Distichodus fasciolatus*	AMNH 253082	t-81-8030	Democratic Republic of Congo	CB, Kasai R.	MT300599	MT301459	MT300664	MT300874	MT301008	MT301099	MT301294	MT301383
*Distichodus fasciolatus*	AMNH 252430	AMCC 197329	Democratic Republic of Congo	CB, Kwilu R., Kikwit.	MT300609	MT301453	MT300673	MT300862	MT301031	MT301100	MT301301	MT301384
*Distichodus fasciolatus*	AMNH 252538	AMCC 197367	Democratic Republic of Congo	CB, Kwilu R.	MT300610	MT301454	MT300674	MT300863	MT301032	MT301101	MT301302	MT301385
*Distichodus fasciolatus*	CU 95835	t-77-7683	Democratic Republic of Congo	CB, Tshopo/Lindi R.	MT300606	MT301448	MT300669	MT300896	MT301009	MT301073	MT301300	MT301386
*Distichodus fasciolatus*	CU 95835	t-77-7684	Democratic Republic of Congo	CB, Tshopo/Lindi R.	MT300611	MT301452	MT300672	MT300864	MT301033	MT301074	MT301305	MT301378
*Distichodus fasciolatus*	CU 92983	t-78-7740	Republic of Congo	CB, Congo R., Bela.	MT300612	MT301455	MT300676	MT300865	MT301026	MT301103	MT301303	MT301347
*Distichodus fasciolatus*	AMNH 240041	t-22-2157	Gabon	LG, Lekoli R.	MT300608	MT301456	MT300667	MT300861	MT301030	MT301096	MT301299	MT301380
*Distichodus fasciolatus*	CU 92982	t-78-7739	Gabon	LG, Lekoli R.	MT300598	MT301449	MT300670	MT300897	MT301035	MT301102	MT301297	MT301346
*Distichodus hypostomatus*	CU 95143	t-78-7741	Gabon	LG, Ngounie R.	MT300531	n/a	n/a	MT300788	MT300976	MT301045	MT301183	MT301416
*Distichodus hypostomatus*	CU 95143	t-77-7685	Gabon	LG, Ngounie R.	MT300532	n/a	n/a	MT300789	MT300977	MT301046	MT301185	MT301417
*Distichodus hypostomatus*	AMNH 249522	t-63-6209	Cameroon	LG, Bitande R.	MT300529	MT301557	MT300725	MT300790	MT300978	MT301047	MT301184	MT301309
*Distichodus hypostomatus*	AMNH 249522	t-63-6210	Cameroon	LG, Bitande R.	MT300530	MT301558	MT300726	MT300791	MT300979	MT301048	MT301186	MT301418
*Distichodus hypostomatus*	AMNH 253909	t-88-8738	Republic of Congo	LG, Niari R.	MT300527	MT301555	MT300723	MT300792	MT300981	MT301049	MT301181	MT301310
*Distichodus hypostomatus*	AMNH 253936	t-88-8787	Republic of Congo	LG, Kouilou R.	MT300528	MT301556	MT300724	MT300793	MT300980	MT301050	MT301182	MT301311
*Distichodus kasaiensis*	AMNH 251295	t-76-7515	Democratic Republic of Congo	CB, Lulua R.	MT300636	MT301474	MT300696	MT300906	MT301002	MT301109	MT301251	MT301392
*Distichodus kasaiensis*	AMNH 243646	t-38-3731	Democratic Republic of Congo	CB, Lulua R.	MT300637	MT301472	MT300697	MT300907	MT301003	MT301110	MT301252	MT301393
*Distichodus kasaiensis*	AMNH 252780	t-80-7914	Democratic Republic of Congo	CB, Lulua R.	MT300638	MT301473	MT300698	MT300908	MT301004	MT301111	MT301250	MT301419
*Distichodus kolleri*	AMNH 249814	t-62-6191	Cameroon	LG, Ebebda	MT300562	n/a	MT300774	MT300814	MT300915	MT301146	MT301187	MT301321
*Distichodus kolleri*	AMNH 249824	t-63-6292	Cameroon	LG	MT300563	n/a	MT300777	MT300825	MT300916	MT301147	MT301188	MT301318
*Distichodus kolleri*	AMNH 249824	t-63-6293	Cameroon	LG	MT300564	n/a	MT300778	MT300826	MT300917	MT301148	MT301189	MT301312
*Distichodus kolleri*	CU 93515	t-77-7686	Cameroon	LG, Djerem R.	MT300565	n/a	MT300775	MT300827	MT300918	MT301149	MT301190	MT301319
*Distichodus kolleri*	AMNH 236538	t-58-5703	Cameroon	LG, Sanaga R.	MT300566	n/a	MT300776	MT300828	MT300919	MT301150	MT301191	MT301320
*Distichodus kolleri*	AMNH 236521	t-58-5718	Cameroon	LG, Sanaga R.	MT300567	n/a	MT300773	MT300829	MT300920	MT301151	MT301192	MT301322
*Distichodus lusosso*	AMNH 256953	t-112-11,160	Democratic Republic of Congo	CB, Pool Malebo.	MT300650	MT301502	MT300702	MT300905	MT301036	MT301083	MT301256	MT301437
*Distichodus lusosso*	AMNH 252809	t-80-7939	Democratic Republic of Congo	CB, Lulua R.	MT300651	MT301499	MT300703	MT300899	MT301038	MT301076	MT301257	MT301431
*Distichodus lusosso*	CU 95830	t-77-7688	Democratic Republic of Congo	CB, Congo R., Wanie-Rukula.	MT300652	MT301497	MT300704	MT300900	MT301039	MT301078	MT301258	MT301433
*Distichodus lusosso*	AMNH 247230	t-50-4901	Democratic Republic of Congo	CB, Lufula R.	MT300654	MT301500	MT300699	MT300902	MT301041	MT301080	MT301254	MT301435
*Distichodus lusosso*	AMNH 250310	t-67-6648	Democratic Republic of Congo	CB, Congo R., Luozi.	MT300655	MT301503	MT300701	MT300903	MT301042	MT301081	MT301255	MT301436
*Distichodus lusosso*	AMNH 250310	t-71-7096	Democratic Republic of Congo	CB, Congo R., Luozi.	MT300649	MT301501	MT300706	MT300904	MT301043	MT301082	MT301260	MT301430
*Distichodus lusosso*	AMNH 240047	t-26-2599	Republic of Congo	CB, Congo River, Mbelo.	MT300653	MT301498	MT300705	MT300901	MT301040	MT301079	MT301259	MT301434
*Distichodus lusosso*	CU 91878	t-77-7687	Central African Republic	CB, Baidou R.	MT300648	MT301496	MT300700	MT300883	MT301037	MT301077	MT301253	MT301432
*Distichodus maculatus*	AMNH 252806	t-80-7944	Democratic Republic of Congo	CB, Lulua R.	MT300524	MT301439	MT300720	MT300911	MT300973	MT301173	MT301178	MT301368
*Distichodus maculatus*	CU 91523	t-77-7690	Central African Republic	Oubangui R., Mobaye.	MT300523	MT301438	MT300719	MT300914	MT300972	MT301174	MT301177	MT301371
*Distichodus maculatus*	CU 95265	t-77-7691	Tanzania	CB, Malagarasi R.	MT300526	MT301440	MT300721	MT300912	MT300975	MT301175	MT301179	MT301369
*Distichodus maculatus*	CU 91120	t-77-7689	Zambia	CB, Luapula R.	MT300525	MT301441	MT300722	MT300913	MT300974	MT301176	MT301180	MT301370
*Distichodus nefasch*	AMNH 264420	AMCC 236881	Ethiopia	NS, Omo R.	MT300595	MT301560	MT300657	MT300910	n/a	MT301115	n/a	n/a
*Distichodus noboli*	AMNH 257170	t-34-3384	Democratic Republic of Congo	CB, Lac Ilungu.	MT300537	MT301526	MT300733	MT300816	MT300926	MT301138	MT301204	MT301376
*Distichodus noboli*	AMNH 247930	t-25-2491	Democratic Republic of Congo	CB, Luilaka R.	MT300533	MT301528	MT300732	MT300817	MT300929	MT301139	MT301198	MT301367
*Distichodus noboli*	AMNH 242501	t-34-3316	Democratic Republic of Congo	CB, Lac Ikenge.	MT300539	MT301529	MT300728	MT300818	MT300924	MT301140	MT301199	MT301364
*Distichodus noboli*	AMNH 241865	t-25-2456	Democratic Republic of Congo	CB, Luilaka R.	MT300536	MT301530	MT300729	MT300819	MT300925	MT301141	MT301200	MT301366
*Distichodus noboli*	AMNH 242502	t-34-3327	Democratic Republic of Congo	CB, Lac Ikenge.	MT300538	MT301527	MT300734	MT300820	MT300927	MT301142	MT301205	MT301377
*Distichodus noboli*	AMNH 249786	t-65-6424	Democratic Republic of Congo	CB, Lomako R.	MT300534	MT301531	MT300730	MT300821	MT300923	MT301143	MT301201	MT301363
*Distichodus noboli*	AMNH 249775	t-65-6412	Democratic Republic of Congo	CB, Maringa R.	MT300535	MT301532	MT300731	MT300822	MT300922	MT301144	MT301202	MT301362
*Distichodus noboli*	AMNH 255007	t-96-9536	Democratic Republic of Congo	CB, Mai-Ndombe R.	MT300540	MT301525	MT300727	MT300823	MT300928	MT301145	MT301203	MT301365
*Distichodus notospilus*	CU 95853	t-78-7743	Gabon	LG, Lekoli R, Gabon	MT300586	MT301554	MT300780	MT300813	MT300968	MT301172	MT301197	MT301324
*Distichodus notospilus*	AMNH 249523	t-63-6213	Cameroon	LG, Bitande R., Cameroon	MT300587	MT301551	MT300781	MT300810	MT300969	MT301167	MT301193	MT301313
*Distichodus notospilus*	AMNH 249537	t-63-6237	Cameroon	LG, Coastal stream.	MT300585	MT301553	MT300779	MT300815	MT300921	MT301168	MT301196	MT301323
*Distichodus notospilus*	AMNH 249523	t-63-6211	Cameroon	LG, Bitande R., Cameroon	MT300588	MT301550	MT300782	MT300811	MT300970	MT301169	MT301194	MT301314
*Distichodus notospilus*	AMNH 249523	t-63-6212	Cameroon	LG, Bitande R., Cameroon	MT300589	MT301552	MT300783	MT300812	MT300971	MT301170	MT301195	MT301315
*Distichodus petersii*	CU 93783	t-77-7693	Tanzania	EC, Kilimbero R.	MT300597	MT301559	MT300656	MT300851	MT300982	MT301044	MT301244	MT301307
*Distichodus rostratus*	AMNH photo voucher	n/a	n/a	NS, aquarium trade	MT300596	MT301561	MT300658	n/a	MT300983	MT301116	MT301245	MT301308
*Distichodus schenga*	SAIAB 97189	RC10C047	Mozambique	Z, Zambezi R.	MT300613	MT301457	n/a	MT300909	MT301013	MT301084	MT301290	MT301379
*Distichodus schenga*	SAIAB 97065	RC10C077	Mozambique	Z, Zambezi R.	MT300614	MT301447	n/a	MT300872	MT301007	MT301085	MT301295	MT301394
*Distichodus sexfasciatus*	AMNH 240874	t-27-2982	Democratic Republic of Congo	CB, Congo R., Bulu.	MT300631	MT301486	MT300679	MT300859	MT300991	MT301063	MT301268	MT301424
*Distichodus sexfasciatus*	AMNH 247254	t-47-4695	Democratic Republic of Congo	CB, Congo R., Bulu.	MT300632	MT301493	MT300681	MT300860	MT300992	MT301070	MT301269	MT301425
*Distichodus sexfasciatus*	AMNH 250133	t-70-6909	Democratic Republic of Congo	CB, Congo R., Luozi.	MT300627	MT301495	MT300686	MT300855	MT300989	MT301066	MT301270	MT301426
*Distichodus sexfasciatus*	AMNH 251085	t-75-7437	Democratic Republic of Congo	CB, Lulua R.	MT300633	MT301488	MT300682	MT300858	MT300995	MT301071	MT301265	MT301427
*Distichodus sexfasciatus*	AMNH 255283	t-100-9953	Democratic Republic of Congo	CB, N’Sele R.	MT300634	MT301487	MT300677	MT300856	MT300990	MT301064	MT301262	MT301428
*Distichodus sexfasciatus*	AMNH 253488	t-83-8276	Democratic Republic of Congo	CB, Congo R., Kinsuka.	MT300635	MT301489	MT300683	MT300857	MT300996	MT301072	MT301263	MT301429
*Distichodus sexfasciatus*	AMNH 251120	t-75-7414	Democratic Republic of Congo	CB, Lulua R.	MT300628	MT301490	MT300684	MT300873	n/a	MT301065	MT301264	MT301420
*Distichodus sexfasciatus*	AMNH 251317	t-76-7552	Democratic Republic of Congo	CB, Lulua R.	MT300629	MT301491	MT300685	MT300852	MT300993	MT301068	MT301266	MT301421
*Distichodus sexfasciatus*	AMNH 240051	t-27-2625	Republic of Congo	CB, Congo R., Mbelo.	MT300626	MT301492	MT300680	MT300854	MT300988	MT301067	MT301261	MT301423
*Distichodus sexfasciatus*	CU 91519	t-77-7694	Central African Republic	CB, Oubangui R.	MT300630	MT301494	MT300678	MT300853	MT300994	MT301069	MT301267	MT301422
*Distichodus teugelsi*	MRAC A7–31-P-348	A8–20#965	Republic of Congo	CB, Lefini R., RC	MT300546	MT301523	MT300745	MT300830	MT300950	MT301117	MT301211	MT301325
*Distichodus teugelsi*	MRAC A7–31-P-349	A7–31#507	Republic of Congo	CB, Lefini R., RC	MT300547	MT301524	MT300746	MT300831	MT300951	MT301118	MT301212	MT301326
*Distichodus teugelsi*	MRAC A8–20-P-210	MRAC 3	Republic of Congo	CB, Lefini R., RC	MT300548	MT301522	MT300747	MT300832	MT300930	MT301119	MT301213	MT301327
*Distichodus teugelsi*	AMNH 253625	t-86-8583	Democratic Republic of Congo	CB, Kwilu R.	MT300543	MT301504	MT300736	MT300848	MT300958	MT301120	MT301206	MT301328
*Distichodus teugelsi*	AMNH 253758	t-87-8607	Democratic Republic of Congo	CB, Kwilu R.	MT300541	MT301505	MT300737	MT300833	MT300959	MT301121	MT301207	MT301329
*Distichodus teugelsi*	AMNH 256221	t-107-10,607	Democratic Republic of Congo	CB, Kwilu R.	MT300542	MT301506	MT300735	MT300850	MT300963	MT301122	MT301208	MT301342

Most tissue samples were obtained from specimens collected during recent expeditions in West and West-Central Africa by a research team from the American Museum of Natural History (AMNH) (led by co-author MLJS). Specimens were handled and euthanized prior to preservation in accordance with recommended guidelines for the use of fishes in research [49] and stress was ameliorated by minimizing handling and through the use of the anesthetic Tricaine mesylate (MS-222) for euthanasia. Tissue samples were taken in the field and immediately preserved in 95% ethanol. Voucher specimens were fixed in formalin and subsequently transferred to 70% ethanol for long-term storage. Data for specimens cataloged and stored in the ichthyology collection of the AMNH, are available online at http://sci-web-001.amnh.org/db/emuwebamnh/index.php.

Specimen collection was made in accordance with ethical and legal guidelines for international animal research approved by the AMNH Institutional Animal Care and Use Committee (IACUC) (approval #36/06). The AMNH IACUC has guidelines relating to studies involving its members in different countries, and this study conforms to those guidelines. Specimen collection and exportation of samples used in this study follow institutional and national ethical and legal guidelines of the Ministry of Fishery and Aquaculture, Republic of Guinea, No. 65/MPA/DGAGSP/11; the Ministry of Scientific Research and Technical Innovation, Republic of Congo, No. 031/MRSIT/DGRST/GERBID.06.13; and the Ministry of Agriculture and Fisheries, Democratic Republic of Congo, No. 037/DP/SG/AGRIPEL/16.

Additional samples were obtained from colleagues at the Cornell University Museum of Vertebrates (CUMV), the Royal Museum for Central Africa (MRAC), and the South African Institute for Aquatic Biodiversity (SAIAB). Voucher specimens are deposited in the ichthyology collections of the AMNH, CUMV, MRAC, and SAIAB. Species identity of non-AMNH vouchers was confirmed either by direct examination of loaned specimens, photographs provided, or on taxonomic authority of the loaning institution. Voucher catalog numbers and GenBank accession numbers for the gene sequences generated and included in this study are listed in Table 3.

### Gene sampling and nucleotide data collection

Eight gene fragments, including the seven protein-coding loci sampled by Arroyave et al. [18] to address distichodontid interrelationships (*co1, cytb, enc1, glyt, myh6, nd2,* and *sh3px3*) were sequenced. Additionally, a faster-evolving mitochondrial non-coding marker, control region (*cr*), was added to address more recent divergences within the genus. DNA sequence data was generated from a total of 133 *Distichodus* individuals. General procedures for DNA extraction, amplification, and purification, along with primers and thermal profiles for sequencing the protein-coding genes used in this study follow Arroyave and Stiassny [50] and Arroyave et al. [18]. *Distichodus*-specific primers for *cr* (cr_Dist_f: 5′-AGCGCCGGTCTTGTAATCCG-3′; cr_Dist_r: 5′-TGCTTGTGGAACTTTCTAGGGTCCAT-3′) were designed using the software Primer3 [51] from conserved flanking regions of aligned mtDNA control region sequences extracted from the two distichodontid complete mitochondrial genomes available in GenBank (*Distichodus sexfasciatus* AB070242 and *Ichthyborus* sp. AP011993). Amplification of *cr* via PCR was carried out using the following thermal profile: 5-min initial denaturation at 95 °C, followed by 35 cycles of denaturation at 95 °C for 60 s, annealing at 58 °C for 60 s, and extension at 72 °C for 120 s, followed by a 10-min final extension at 72 °C.

### Sequence editing and partitioning scheme/substitution model selection

Contig assembly and sequence editing was performed using Geneious v.11.0.2 [52]. IUPAC nucleotide ambiguity codes were used to represent heterozygous sites. The resulting sequences were trimmed to exclude primer regions and examined for appropriateness/homology using BLASTx [53]. Each gene was aligned using MUSCLE [54] under default parameters as implemented in Geneious, followed by concatenation of individual alignments. All sequences were checked for stop codons and for miscalled amino acids by examining translation alignments.

Best-fit partitioning schemes and models of molecular evolution for the nucleotide data were determined using PartitionFinder2 [55] based on 22 pre-defined data blocks: the non-coding mtDNA control region (1 block) plus the 1st, 2nd, and 3rd codon positions of the seven protein-coding genes (3 positions × 7 genes). The PartitionFinder2 greedy algorithm was employed to search for an optimal scheme under the assumption of independent model parameters and branch lengths for each partition. Selection of the partitioning scheme and models over the set of schemes and models produced during greedy search was accomplished using the Schwarz/Bayesian Information Criterion (BIC) [56].

### Phylogenetic, biogeographic, and chronological analyses

Various analytical approaches were employed to infer phylogenetic relationships in *Distichodus* from the multilocus dataset generated in this study, one of which also simultaneously estimates absolute times of divergence in the resultant phylogeny. The results from the latter approach were subsequently used in analyses for testing historical biogeographic hypotheses of geographic range evolution in *Distichodus*.

#### Maximum likelihood (ML) estimation of phylogeny

Phylogenetic analysis of the concatenated alignment of the eight sampled genes under a Total Evidence/Simultaneous Analysis [57, 58] approach was performed using the ML optimality criterion. Furthermore, to examine the degree of variation in topology, resolution, and clade support among the individual sampled loci, and to complement the inferences made from the simultaneous analysis of all markers, each of the nuclear genes (*enc1*, *glyt*, *myh6*, *sh3px3*) and a concatenated alignment of the mitochondrial genes (*co1*, *cr*, *cytb*, *nd2*; effectively inherited as a single locus), were independently analyzed, also using the ML optimality criterion. ML phylogenetic analyses were conducted with RAxML v.8 [59] through the CIPRES Science Gateway v.3.3 [60] as a single partition under the GTRGAMMA model with four rate classes using full ML optimization for the tree search and 1000 rapid bootstrap (BS) searches to assess nodal support [61].

#### Species-tree approaches

Although concatenation methods have been suggested to often perform well when incomplete lineage sorting (ILS) levels are low [24], the degree of ILS in *Distochodus* is unknown. To explore the outcomes of ILS-aware species-tree analyses relative to concatenation, both SVDquartets [62] and ASTRAL-III [63] were employed. SVDquartets has been suggested to perform well with low ILS and small numbers of sites per gene, and ASTRAL methods have been suggested to perform well under high ILS conditions, but may be sensitive to small numbers of sites per gene [24]. SVDquartets analysis was conducted in PAUP* v4.0a164 [64] sampling all ~ 8.6 million quartets under the multispecies coalescent on the full dataset, using the default QFM quartet assembly method. Bootstrap support values were assembled onto the SVDquartets tree using the *sumtrees* command in the DendroPy package [65]. Gene trees input to ASTRAL-III were estimated from best-fit codon models inferred in codonPhyML [66] under default search intensity, using custom R scripts written by the authors. Because the mitochondrial genome does not undergo recombination and is inherited as a single locus, the three protein-coding mitochondrial genes were fit with a single codon model and inferred gene tree. Gene trees for each autosomal locus were inferred separately.

### Bayesian co-estimation of phylogeny and divergence times

Prior to co-estimation of phylogeny and divergence times, a new data matrix was created from the original multi-individual, multi-locus matrix, by including DNA sequence data from only a single individual per species, from or near the type locality whenever possible (for each sampled species, the first individual listed in Table 3). The resulting reduced matrix was analyzed in BEAST v.2.5.0 [67] under the optimal partitioning scheme and substitution models suggested by the PartitionFinder2 analysis. Node ages were estimated using a Bayesian relaxed-clock method [68] under the uncorrelated lognormal (UCLN) rate variation model, and assuming a birth-death process prior for topology and divergence times. By default, the prior on the mean parameter of the UCLN clock model (*ucldMean.*c) is a uniform distribution on the interval (0*,*∞), which is an uninformative and improper prior (it does not integrate to 1). Although improper priors can sometimes lead to proper posterior distributions, they may also have undesired effects and cause problems with mixing and convergence [69]. Based on previous findings regarding substitution rates in *Distichodus* [18], we assumed a log-normally distributed prior for the clock rate (*ucldMean.*c) with hyperparameters μ = 0.003 and σ = 0.5. On the other hand, the standard deviation parameter of the UCLN clock model (*ucldStdev.c*) is by default assigned a gamma distribution prior. Variation in substitution rates among branches in *Distichodus*, however, appears to be low in general [18]. Accordingly, we assumed an exponential prior distribution with 95% of the probability density on values < 1 for the standard deviation of the UCLN (*ucldStdev.c*).

**Fig. 8:**
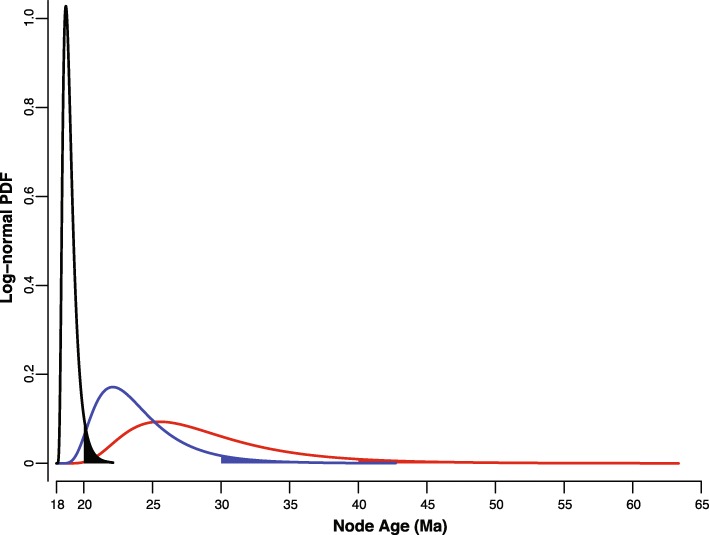
Alternative log-normally distributed priors used to account for temporal uncertainty of calibration nodes. Each prior probability density function (PDF) is characterized by a hard minimum bound of 18 Ma (set by the age of the calibration fossil), a standard deviation (σ) of 0.5, and a variable mean (μ) (in real space) that probabilistically models the extent to which the node age spreads into the past: μ = 19 (black), μ = 24 (blue), and μ = 29 (red). The lower limit of the x-axis interval defining the area shaded under each curve corresponds to its 95th percentile soft maximum bound (*P*_*95*_ SMB): 20 Ma (black), 30 Ma (blue), and 40 Ma (red)

The molecular clock was calibrated based on early Miocene (ca. 18 Ma) fossilized dentition attributable to *Distichodus* recovered from deposits of the Maradah Formation in Jabal Zaltan, Libya, by far the oldest fossil unambiguously assignable to the genus [35]. In fact, this fossil pushes back the first known appearance of *Distichodus* in the fossil record by 10 Ma with respect to the *Distichodus* calibration fossil used by Arroyave et al. [18] to infer a time-scaled phylogeny of citharinoid fishes. Although the Maradah fossil is unquestionably diagnostic of *Distichodus* (tall, slender necked tooth with a bifid apex bearing characteristically short and rounded lobes) and could potentially be ascribed to either *Distichodus nefasch* or *D. rostratus* on the basis of size and geographic distribution, its exact phylogenetic placement is unknown. The absence of relevant comparative morphological data in a phylogenetic context to which to integrate the fossil taxon, coupled with its fragmentary nature, renders it difficult to confidently assign it to a particular node and to determine whether it should be used to constrain the age of the stem or the crown group of the calibration node. Because of this phylogenetic uncertainty, along with the challenge of objectively establishing a maximum age constraint to the calibration node, we conducted a series of analyses (Table 4) to assess the robustness of node ages to analytical ambiguity and to offer alternative output scenarios based on a variety of reasonable input parameters, particularly with respect to the phylogenetic placement of the calibration node and its maximum age constraint. Specifically, we used three alternative calibration nodes: 1) MRCA of *Distichodus* and *Paradistichodus* (D + P), 2) MRCA of *Distichodus* (D), and 3) MRCA of *D. nefasch* and *D. rostratus* (D_ne_ + D_ro_)*.* The rationale behind this proposal is that, at the very least, the calibration fossil could be used to constrain the age of divergence between *Distichodus* and its sister group, *Paradistichodus*, but under more liberal phylogenetic designations, it could also be used to constrain the age of the entire genus or even the divergence between the species *D. nefasch* and *D. rostratus*. Furthermore, each calibration node was constrained both as stem and as crown group. Additionally, the temporal uncertainty of calibration nodes was modeled using log-normally distributed priors with a hard minimum bound set by the age of the fossil (18 Ma) and one of three alternative 95th percentile soft maximum bounds (*P*_*95*_ SMBs): 20, 30, and 40 Ma (Fig. 8; Table 4). The combinatorial exercise of choosing one of three alternative calibration nodes, constrained as stem or crown, and modeled by a log-normally distributed prior characterized by one of three alternative *P*_*95*_ SMBs, resulted in 18 different analyses (although effectively 15 since the node representing the MRCA of *Distichodus* as stem is equivalent to the node representing the MRCA of *Distichodus* and *Paradistichodus* as crown (Table 4). In each analysis, root age was indirectly constrained (as an implied prior) by the combined effects of the calibration prior on other internal node and the prior for topology and divergence times (birth-death process).

**Table 4:** Alternative BEAST2 analyses (1–15) for co-estimating phylogeny and divergence times in *Distichodus* resulting from variable calibration strategies (calibration node, stem vs. crown group, and 95th percentile [*P*_*95*_] soft maximum bound [SMB] of calibration prior)

Calibration node		Lognormal PDF ***P***_***95***_ SMB		
		20 Ma	30 Ma	40 Ma
MRCA of *Distichodus* & *Paradistichodus*	Stem	1	2	3
	Crown	4	5	6
MRCA of *Distichodus*	Crown	7	8	9
MRCA of *D. nefasch* & *D. rostratus*	Stem	10	11	12
	Crown	13	14	15

BEAST2 analyses were implemented using the Markov Chain Monte Carlo algorithm (MCMC) run for 50 million generations sampled every 1000 generations, under default proposal mechanisms and default priors for the parameters of the birth-death branching process used to provide the prior distribution for the non-calibration nodes (speciation and extinction rates) and the model of molecular evolution for each gene (substitution rates, base frequencies, gamma shape, and proportion of invariant sites). Convergence model parameter estimates were assessed via ESS values over 200, using Tracer v.1.7 [70]. Sufficient sampling of the estimate of the tree topology (ESS > 200) was determined by dividing the topological approximate ESS by the generation number of the approximate earliest stationary value in the topological autocorrelation plot, generated in the R package *rwty* [71]. Further assessment of MCMC convergence was undertaken by examination of the average standard deviation of split frequencies, with values << 0.01 taken as indicative of stationarity. All analyses used a 10% burn-in. A maximum clade credibility (MCC) topology was inferred using TreeAnnotator v.2.5 [67], resulting in a chronogram indicating posterior probabilities (PP) and mean ages of all nodes with their associated 95% highest posterior density (HPD) intervals.

#### Inference of geographic range evolution

The evolution of geographic ranges in *Distichodus* was investigated using the null-range-excluded dispersal-extinction-cladogenesis model (DEC*) [72], a modified version of the original likelihood-based dispersal-extinction-cladogenesis (DEC) model [73, 74]. The set of discrete geographic areas for the DEC* analysis consisted of the six Afrotropical ichthyofaunal provinces of Roberts [1] (modified by Lévêque [2]) with presence of *Distichodus* species: Congo Basin (CB), Zambezi (Z), Nilo-Sudan (NS), Upper Guinea (UG), Lower Guinea (LG), and East Coast (EC) (Fig. 1). African ichthyofaunal provinces were delimited on the basis of current and historical patterns of drainage connectivity and the composition of the fish fauna, and therefore represent regions with a distinctive evolutionary history and a more or less characteristic biota at the species and higher taxonomic levels [1, 2]. To assess the relative fits of alternative models of faunal assemblage in the Congo Basin, three variants of the DEC* model were fit to the data in the *BioGeoBEARS* R package [75], following the parameterization of dispersal multipliers from Day et al. [38]: **M0**, an unconstrained multiplier matrix allowing for dispersal to and from the Congo Basin; **M1**, an asymmetric multiplier matrix allowing only dispersal out of the Congo Basin (CB-as-source); **M2**, an asymmetric multiplier matrix allowing only dispersal into the Congo Basin (CB-as-sink). Tip-state ranges were assigned based on the presence of species in different ichthyofaunal provinces. In several cases, species spanned multiple provinces. The maximum range size was set to widespread (all six ichthyofaunal provinces). Given the high dimensionality of the transition matrix resulting from the combination of different provinces (areas) into ranges of sizes up to six, relative to the size of the dataset, 14 disjunct ranges of differing sizes were pruned from analysis, reducing the dimensionality of the matrix from 64 × 64 to 50 × 50. To assess the stability of numerical optimization, analysis was run five times from fresh R sessions. Model fits of the M0, M1, and M2 variants were compared using the Akaike information criterion [76] and supports were assessed using Akaike weights [77]. In an effort to take account of chronological uncertainty due to alternative molecular clock calibration scenarios, inference of geographic range evolution in *Distichodus* was conducted on three of the 15 time-scaled phylogenies previously inferred with BEAST2, namely the chronograms resulting from analyses based on each alternative calibration node constrained as crown and by a relatively moderate soft maximum bound (*P*_*95*_ SMB = 30 Ma) (analyses 5, 8, and 14 in Table 4).

## Supplementary information

**Additional file 1: Figure S1.***enc1 Distichodus* phylogeny as inferred by likelihood in RAxML. Colored circles on nodes indicate degree of clade support as determined by bootstrap values (BS). The identity of leaves (terminals) not printed on the tree is specified by the species name (in bold) at the base of the most recent labeled ancestral node from which the sample descends. Names in bold black correspond to those species resolved as monophyletic (when multiple individuals were available), whereas those in bold green indicate that, while most of the sampled specimens fall into the clade subtended by that node, some samples fall outside the clade, and therefore the species is not resolved as monophyletic. Outgroup taxon (*Paradistichodus dimiatus*) not shown.

**Additional file 2: Figure S2.***glyt Distichodus* phylogeny as inferred by likelihood in RAxML. Same contextual information as in Fig. S1.

**Additional file 3: >Figure S3.**. *myh6 Distichodus* phylogeny as inferred by likelihood in RAxML. Same contextual information as in Fig. S1.

**Additional file 4: Figure S4.***sh3px3 Distichodus* phylogeny as inferred by likelihood in RAxML. Same contextual information as in Fig. S1.

**Additional file 5: Figure S5.** mtDNA (*co1, cr, cytb, nd2*) *Distichodus* phylogeny as inferred by likelihood in RAxML. Same contextual information as in Fig. S1.

**Additional file 6: Figure S6.** A time-scaled phylogeny of *Distichodus*. Chronogram resulting from BEAST2 analysis 1. Same contextual information as in Fig. 6.

**Additional file 7: Figure S7.** A time-scaled phylogeny of *Distichodus*. Chronogram resulting from BEAST2 analysis 2. Same contextual information as in Fig. 6.

**Additional file 8: Figure S8.** A time-scaled phylogeny of *Distichodus*. Chronogram resulting from BEAST2 analysis 3. Same contextual information as in Fig. 6.

**Additional file 9: Figure S9.**  A time-scaled phylogeny of *Distichodus*. Chronogram resulting from BEAST2 analysis 4. Same contextual information as in Fig. 6.

**Additional file 10: Figure S10.** A time-scaled phylogeny of *Distichodus*. Chronogram resulting from BEAST2 analysis 5. Same contextual information as in Fig. 6.

**Additional file 11: Figure S11.**  A time-scaled phylogeny of *Distichodus*. Chronogram resulting from BEAST2 analysis 6. Same contextual information as in Fig. 6.

**Additional file 12: Figure S12.** A time-scaled phylogeny of *Distichodus*. Chronogram resulting from BEAST2 analysis 7. Same contextual information as in Fig. 6.

**Additional file 13: Figure S13.** A time-scaled phylogeny of *Distichodus*. Chronogram resulting from BEAST2 analysis 9. Same contextual information as in Fig. 6.

**Additional file 14: Figure S14.** A time-scaled phylogeny of *Distichodus*. Chronogram resulting from BEAST2 analysis 10. Same contextual information as in Fig. 6.

**Additional file 15: Figure S15.** A time-scaled phylogeny of *Distichodus*. Chronogram resulting from BEAST2 analysis 11. Same contextual information as in Fig. 6.

**Additional file 16: Figure S16.** A time-scaled phylogeny of *Distichodus*. Chronogram resulting from BEAST2 analysis 12. Same contextual information as in Fig. 6.

**Additional file 17: Figure S17.** A time-scaled phylogeny of *Distichodus*. Chronogram resulting from BEAST2 analysis 13. Same contextual information as in Fig. 6.

**Additional file 18: Figure S18.** A time-scaled phylogeny of *Distichodus*. Chronogram resulting from BEAST2 analysis 14. Same contextual information as in Fig. 6.

**Additional file 19: Fig. S19.** A time-scaled phylogeny of *Distichodus*. Chronogram resulting from BEAST2 analysis 15. Same contextual information as in Fig. 6.

**Additional file 20: Figure S20.** A spatiotemporal reconstruction of *Distichodus* range evolution. Based on the optimal DEC* model (M1; CB-as-source) and input chronogram resultant from BEAST2 analysis 5. Ichthyofaunal provinces color-coded and abbreviated as in Fig. 1. Probabilities of ancestral areas at each node are presented in Table S2.

**Additional file 21: Figure S21.** A spatiotemporal reconstruction of *Distichodus* range evolution. Based on the optimal DEC* model (M1; CB-as-source) and input chronogram resultant from BEAST2 analysis 14. Ichthyofaunal provinces color-coded and abbreviated as in Fig. 1. Probabilities of ancestral areas at each node are presented in Table S3.

**Additional file 22: Table S1.** Probabilities of ancestral states/ranges at each node of the spatiotemporal reconstruction of *Distichodus* range evolution presented in Fig. 7**.** Columns indicate ancestral areas, represented by all unique combinations for all possible group sizes for the six ichthyofaunal provinces. Rows indicate nodes, with numbering following the typical R phylo format, i.e., 1 is the first tip taxon/area, beginning at the bottom. After the last tip value, the numbering begins at the root, and moves tipward. Ichthyofaunal provinces abbreviated as in Fig. 1.

**Additional file 23: Table S2.** Probabilities of ancestral states/ranges at each node of the spatiotemporal reconstruction of *Distichodus* range evolution presented in Fig. S20. Same contextual information as in Table S1.

**Additional file 24: Table S3.** Probabilities of ancestral states/ranges at each node of the spatiotemporal reconstruction of *Distichodus* range evolution presented in Fig. S21. Same contextual information as in Table S1.

## Data Availability

The DNA sequence data supporting the results of this article are available in the GenBank® repository (http://www.ncbi.nlm.nih.gov) under accession numbers MT300523-MT301561 (see Table 3). Voucher specimens are deposited and readily available in their respective ichthyology collections.

## Abbreviations

AMCC: Ambrose Monell Cryo Collection
AMNH: American Museum of Natural History
BS: Bootstrap
CB: Congo Basin
CUMV: Cornell University Museum of Vertebrates
DEC: Dispersal-extinction-cladogenesis
EC: East Coast
ESS: Effective Sample Size
HPD: Highest Posterior Density
LG: Lower Guinea
ML: Maximum Likelihood
MCMC: Markov chain Monte Carlo
MCC: Maximum clade credibility
NS: Nilo-Sudan
PP: Posterior probabilities
MRAC: Royal Museum for Central Africa
BIC: Schwarz/Bayesian Information Criterion
SAIAB: South African Institute for Aquatic Biodiversity
UCLN: Uncorrelated lognormal
UG: Upper Guinea
Z: Zambezi
