# Supplementary figures and images for: Pattern and timing of diversification in the African freshwater fish genus Distichodus (Characiformes: Distichodontidae)
Source: BMC Evol Biol. 2020 Apr 26;20:48. doi: 10.1186/s12862-020-01615-6 (PMC7184684; doi:10.1186/s12862-020-01615-6)

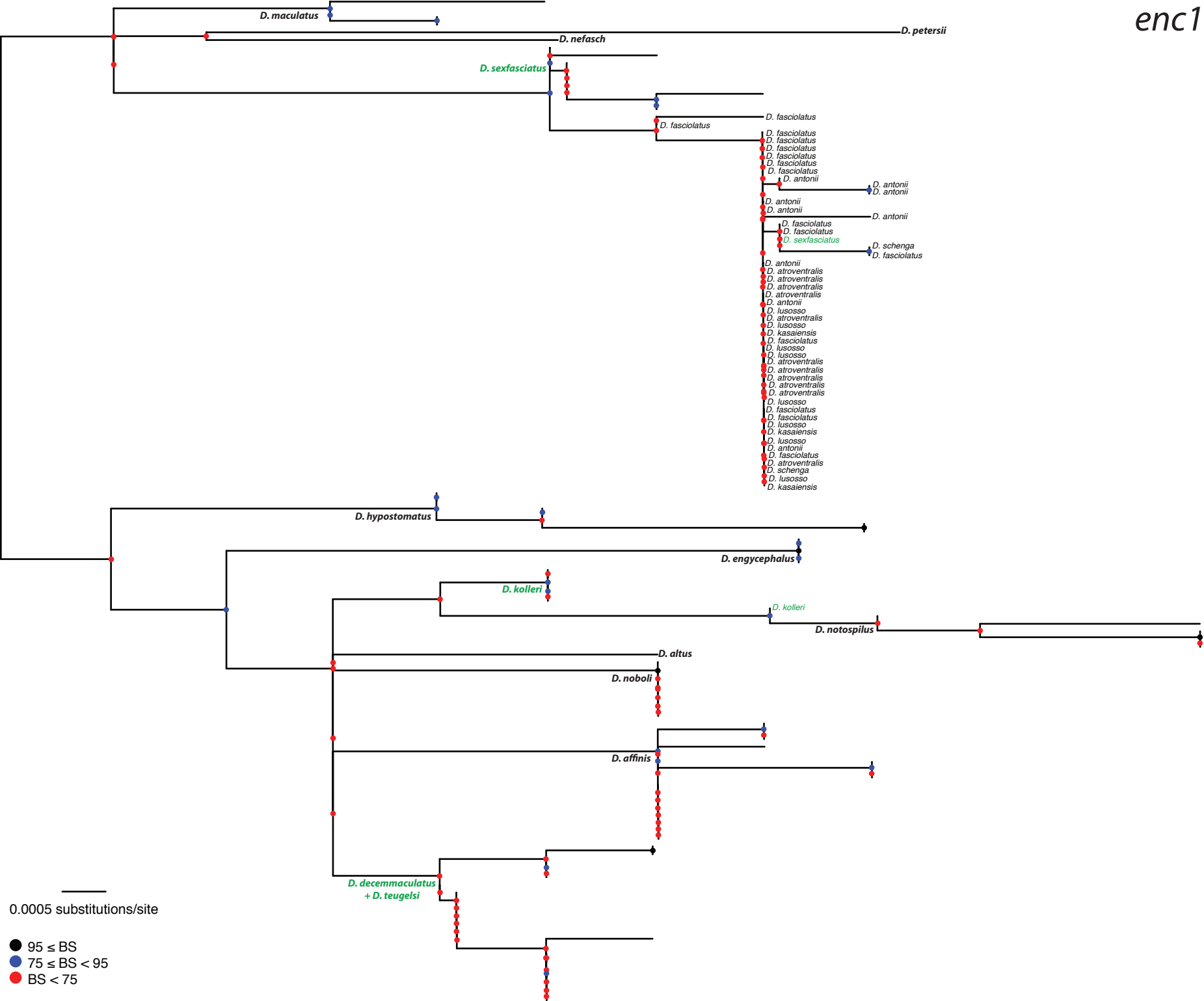

Supplement: Supplementary file 1 — Additional file 1: Figure S1.enc1 Distichodus phylogeny as inferred by likelihood in RAxML. Colored circles on nodes indicate degree of clade support as determined by bootstrap values (BS). The identity of leaves (terminals) not printed on the tree is specified by the species name (in bold) at the base of the most recent labeled ancestral node from which the sample descends. Names in bold black correspond to those species resolved as monophyletic (when multiple individuals were available), whereas those in bold green indicate that, while most of the sampled specimens fall into the clade subtended by that node, some samples fall outside the clade, and therefore the species is not resolved as monophyletic. Outgroup taxon (Paradistichodus dimiatus) not shown. [file 12862_2020_1615_MOESM1_ESM.pdf]

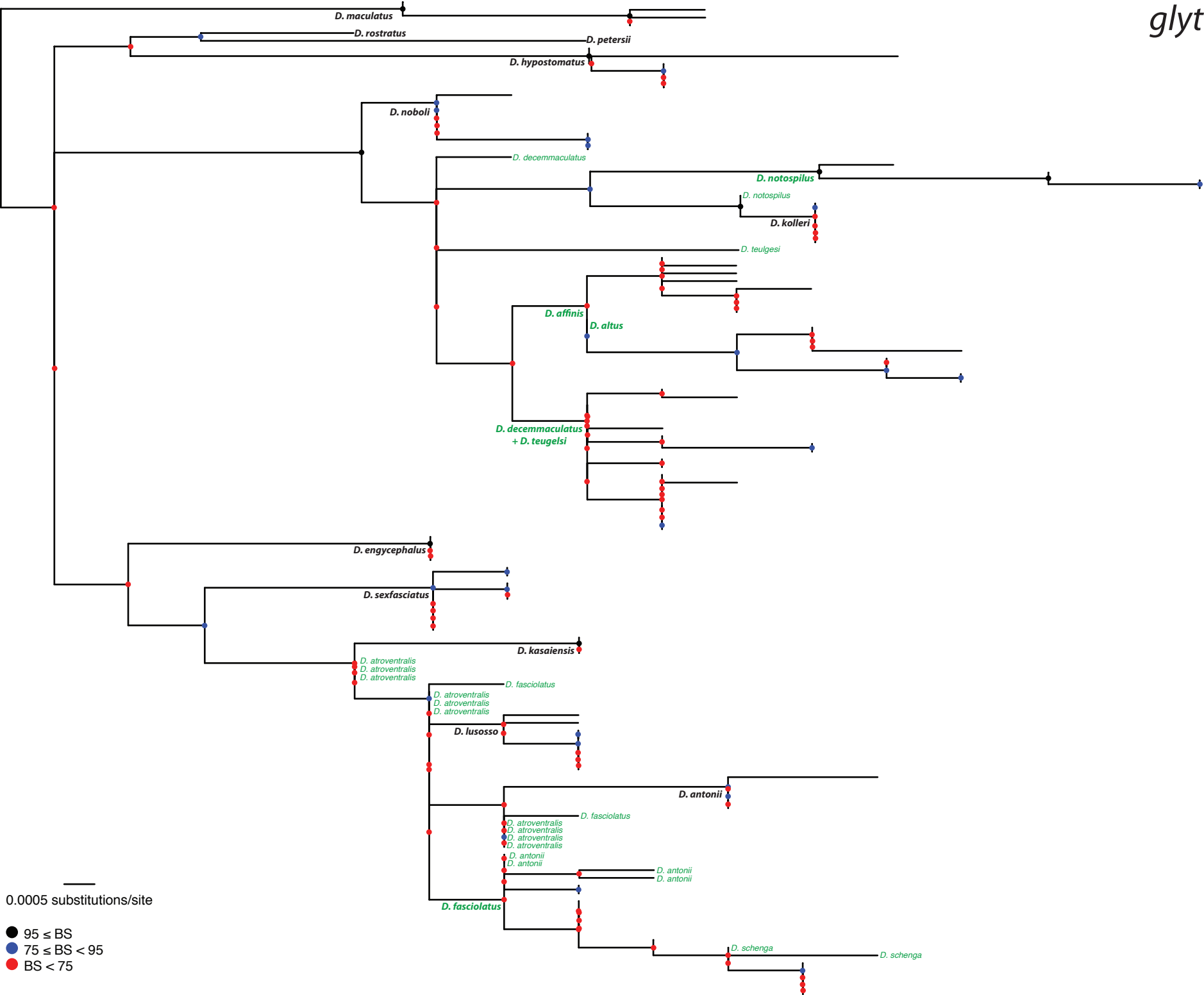

Supplement: Supplementary file 2 — Additional file 2: Figure S2.glyt Distichodus phylogeny as inferred by likelihood in RAxML. Same contextual information as in Fig. S1. [file 12862_2020_1615_MOESM2_ESM.pdf]

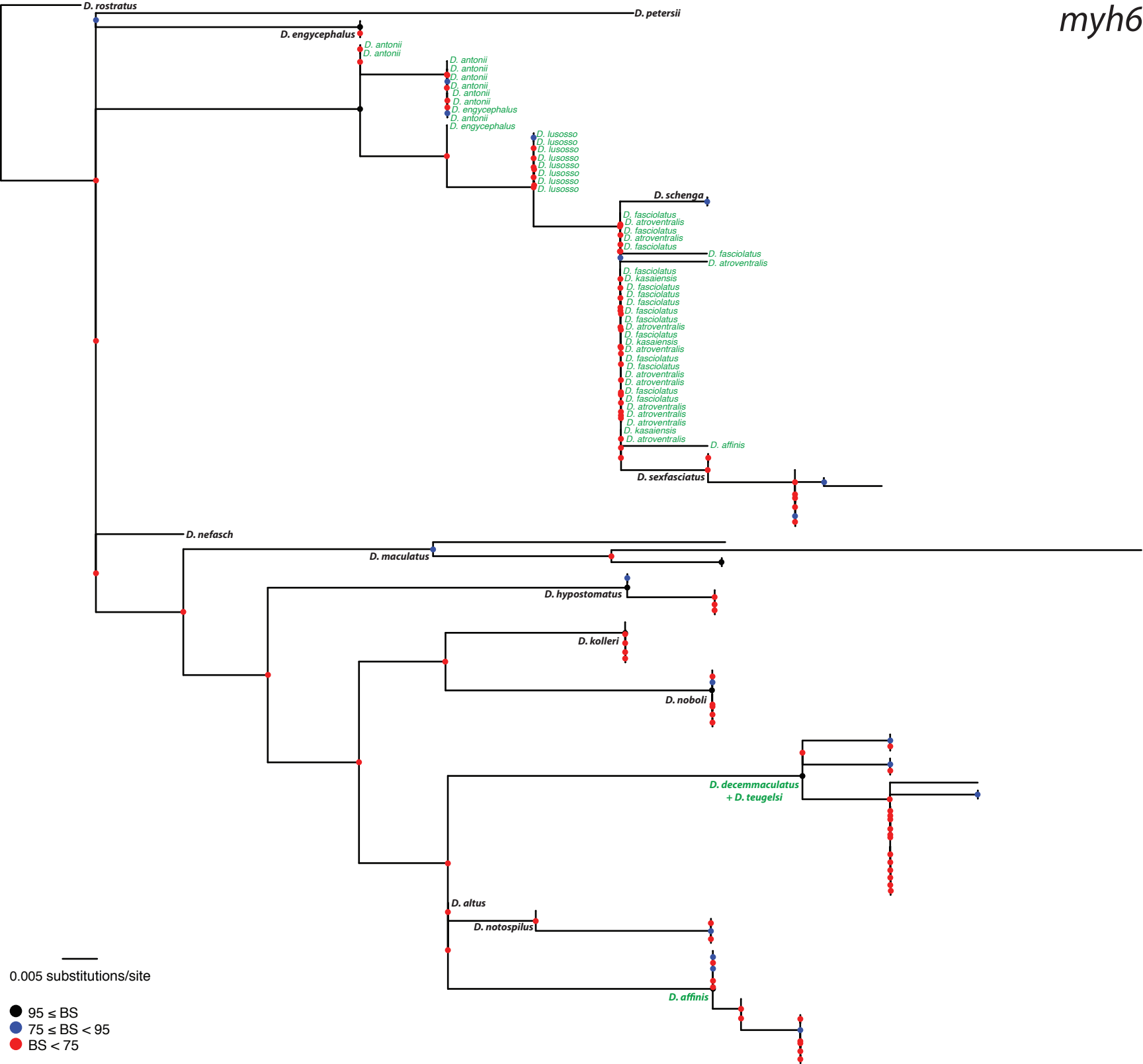

Supplement: Supplementary file 3 — Additional file 3: >Figure S3.. myh6 Distichodus phylogeny as inferred by likelihood in RAxML. Same contextual information as in Fig. S1. [file 12862_2020_1615_MOESM3_ESM.pdf]

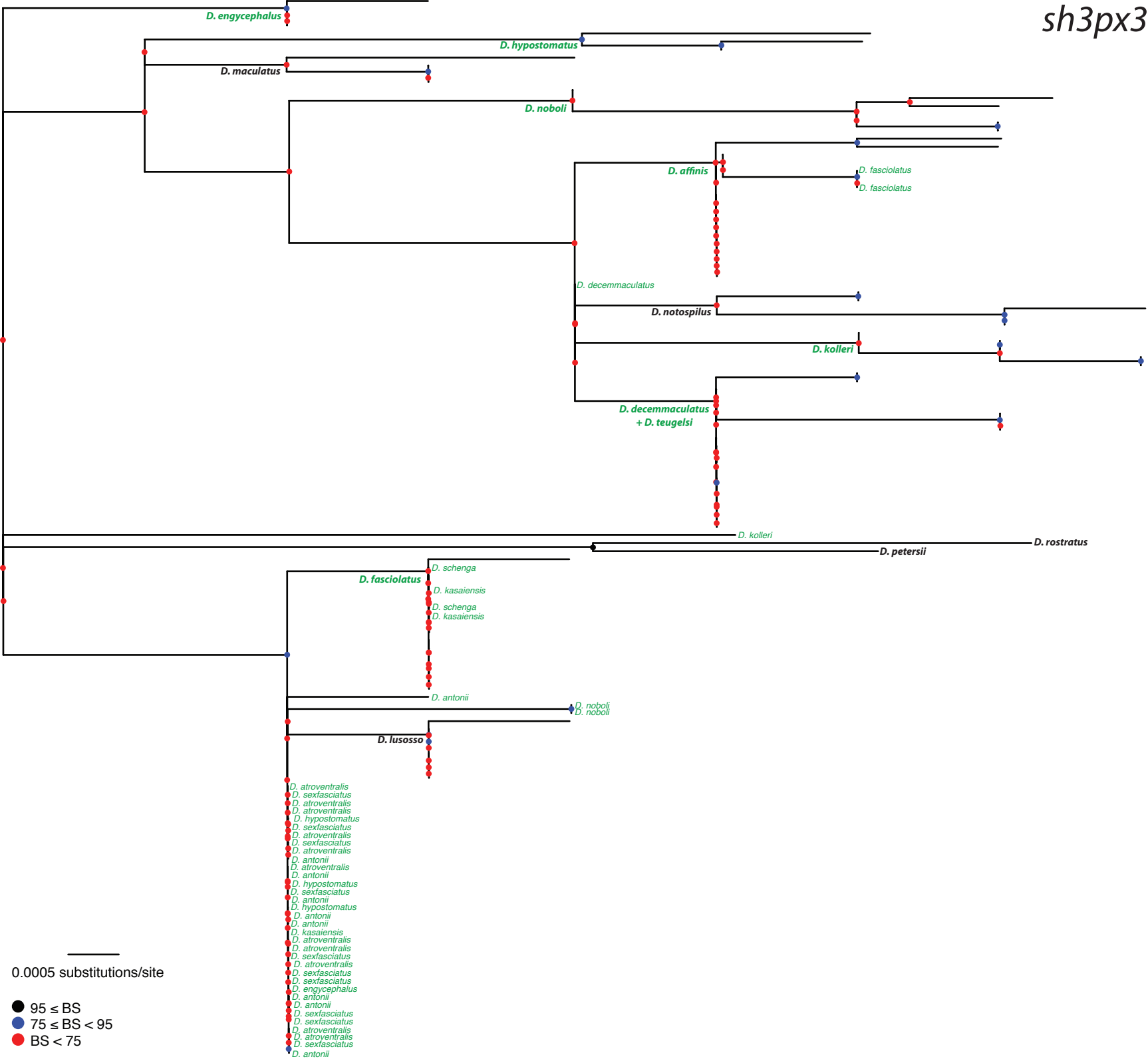

Supplement: Supplementary file 4 — Additional file 4: Figure S4.sh3px3 Distichodus phylogeny as inferred by likelihood in RAxML. Same contextual information as in Fig. S1. [file 12862_2020_1615_MOESM4_ESM.pdf]

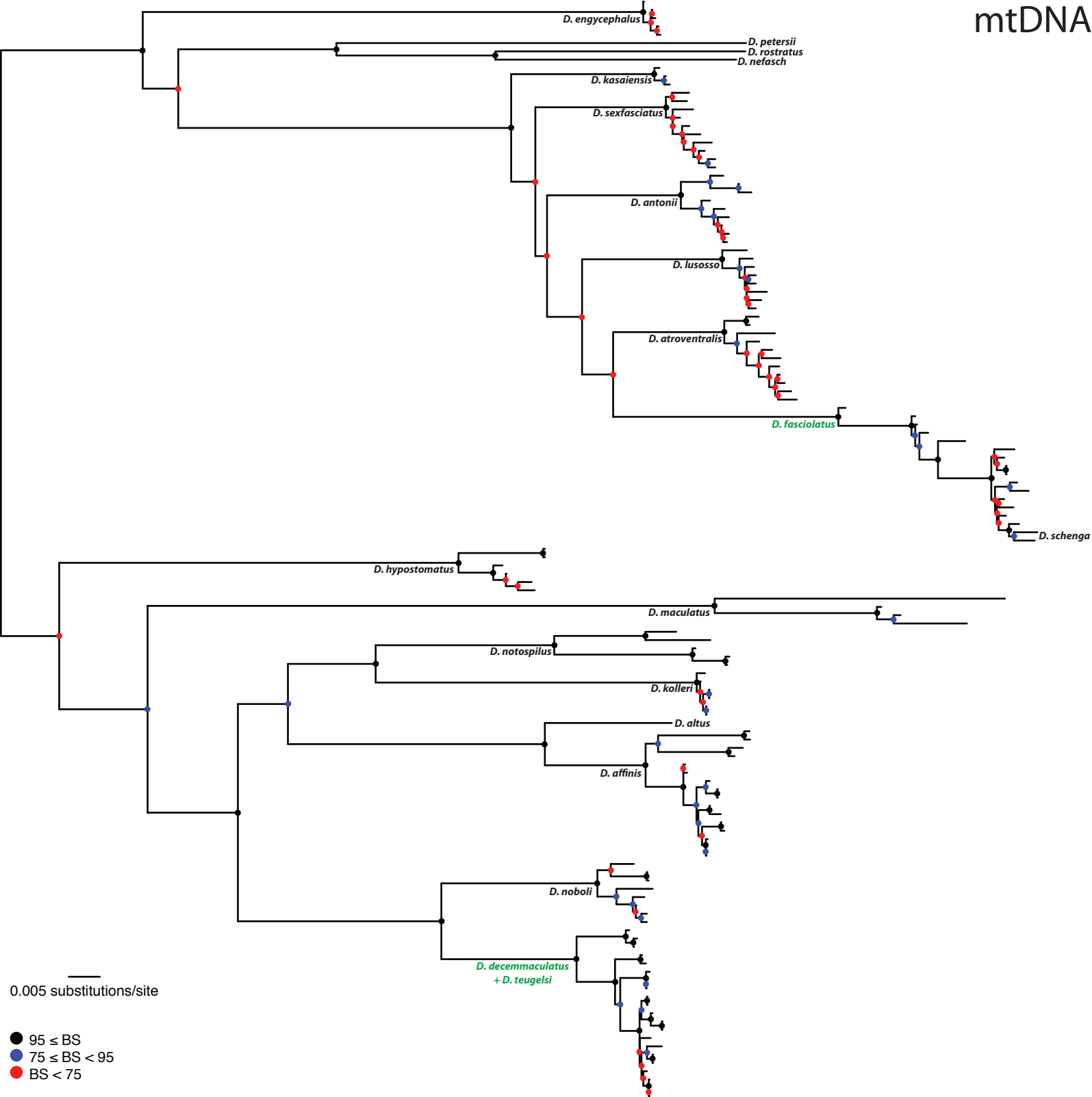

Supplement: Supplementary file 5 — Additional file 5: Figure S5. mtDNA (co1, cr, cytb, nd2) Distichodus phylogeny as inferred by likelihood in RAxML. Same contextual information as in Fig. S1. [file 12862_2020_1615_MOESM5_ESM.pdf]

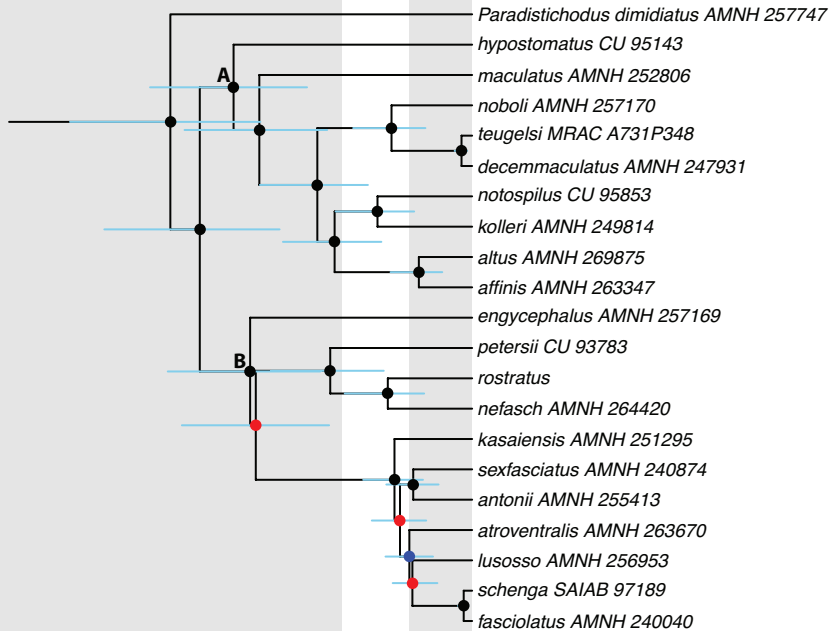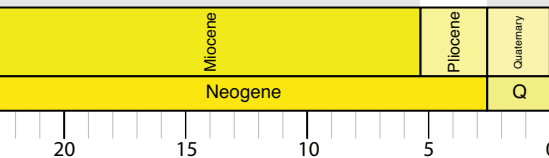

Supplement: Supplementary file 6 — Additional file 6: Figure S6. A time-scaled phylogeny of Distichodus. Chronogram resulting from BEAST2 analysis 1. Same contextual information as in Fig. 6. [file 12862_2020_1615_MOESM6_ESM.pdf]

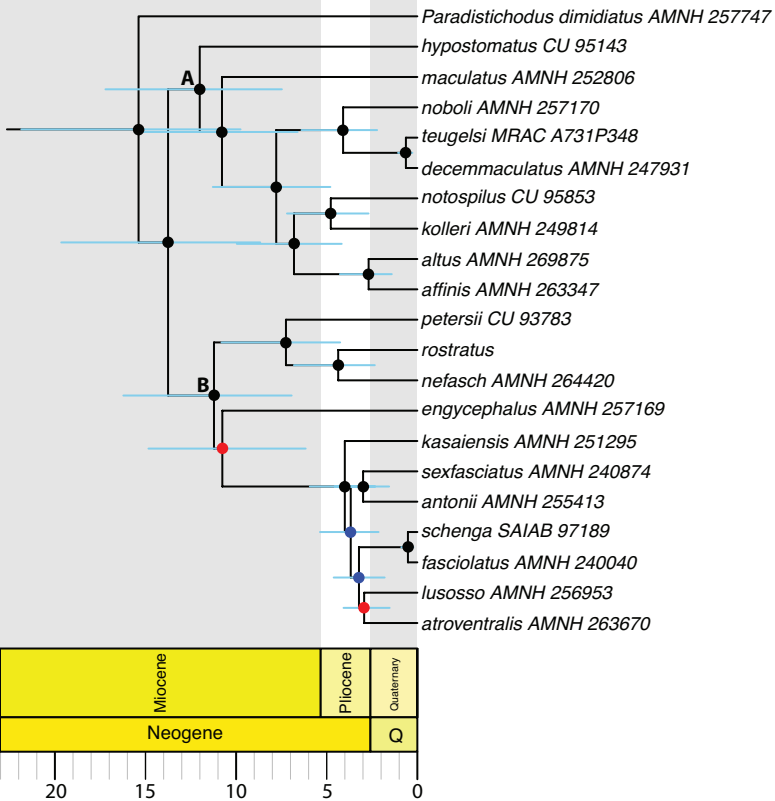

Supplement: Supplementary file 7 — Additional file 7: Figure S7. A time-scaled phylogeny of Distichodus. Chronogram resulting from BEAST2 analysis 2. Same contextual information as in Fig. 6. [file 12862_2020_1615_MOESM7_ESM.pdf]

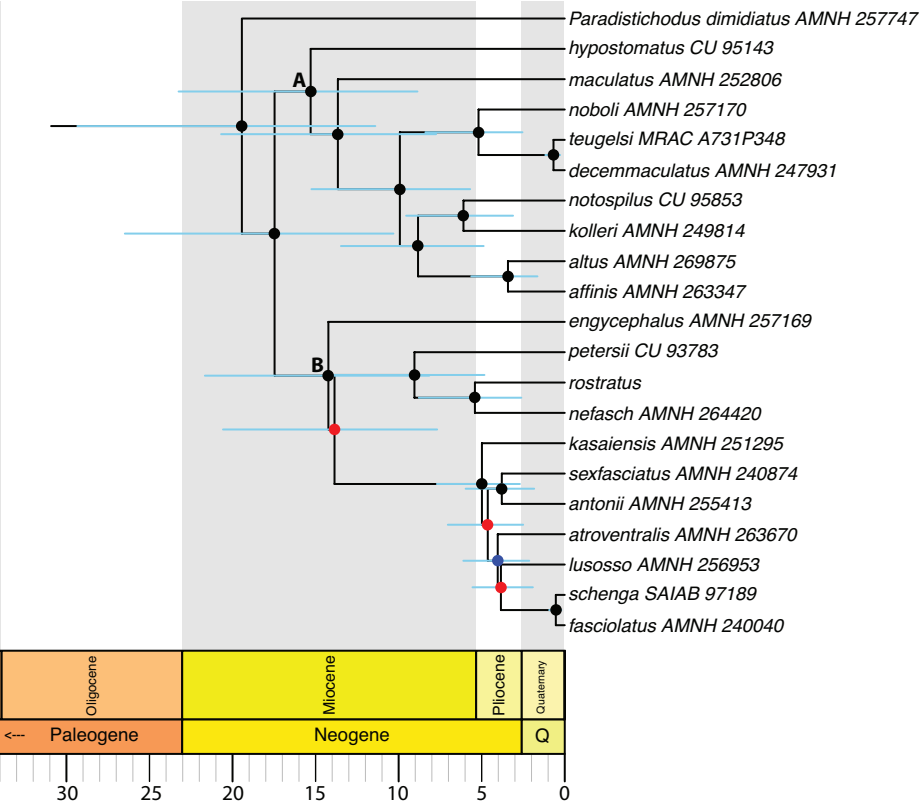

Supplement: Supplementary file 8 — Additional file 8: Figure S8. A time-scaled phylogeny of Distichodus. Chronogram resulting from BEAST2 analysis 3. Same contextual information as in Fig. 6. [file 12862_2020_1615_MOESM8_ESM.pdf]

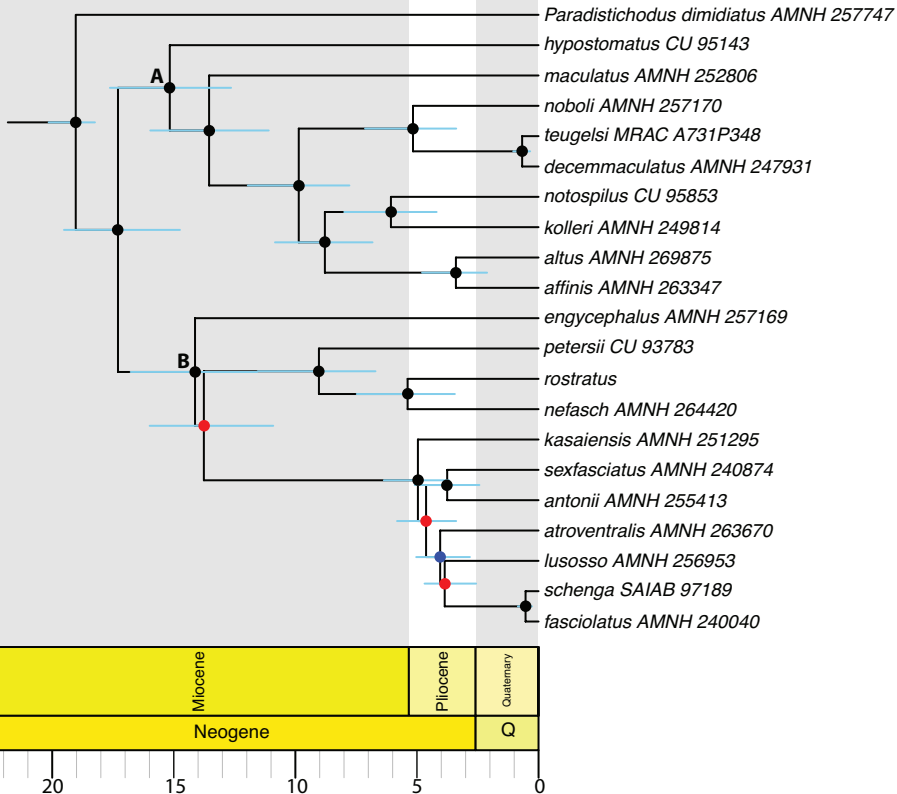

Supplement: Supplementary file 9 — Additional file 9: Figure S9. A time-scaled phylogeny of Distichodus. Chronogram resulting from BEAST2 analysis 4. Same contextual information as in Fig. 6. [file 12862_2020_1615_MOESM9_ESM.pdf]

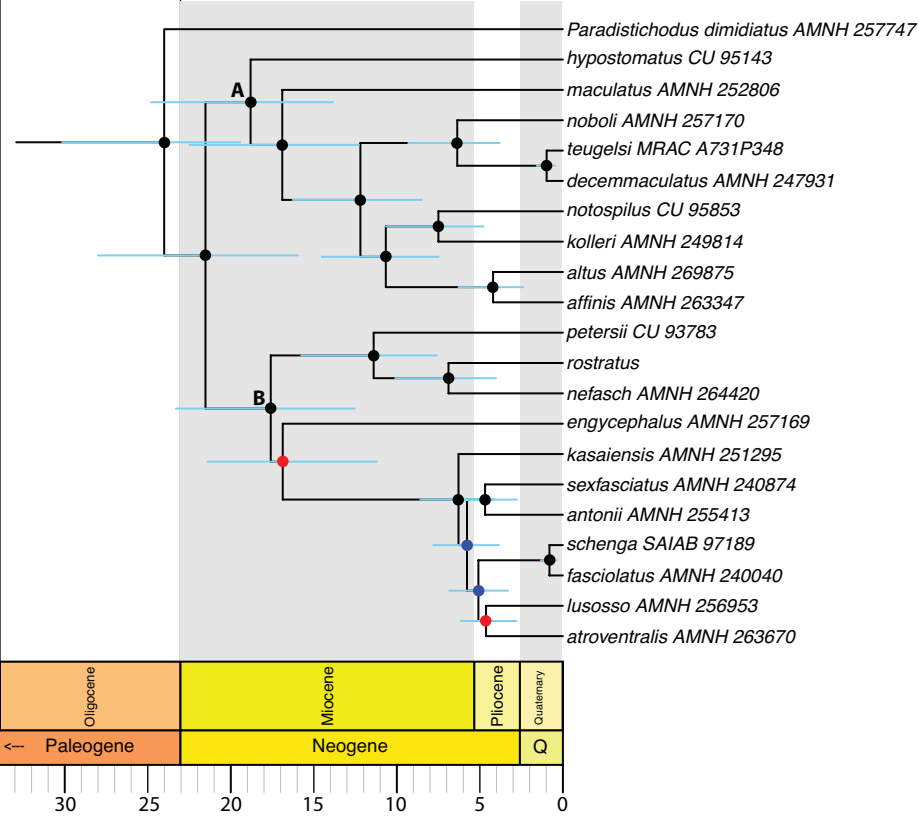

Supplement: Supplementary file 10 — Additional file 10: Figure S10. A time-scaled phylogeny of Distichodus. Chronogram resulting from BEAST2 analysis 5. Same contextual information as in Fig. 6. [file 12862_2020_1615_MOESM10_ESM.pdf]

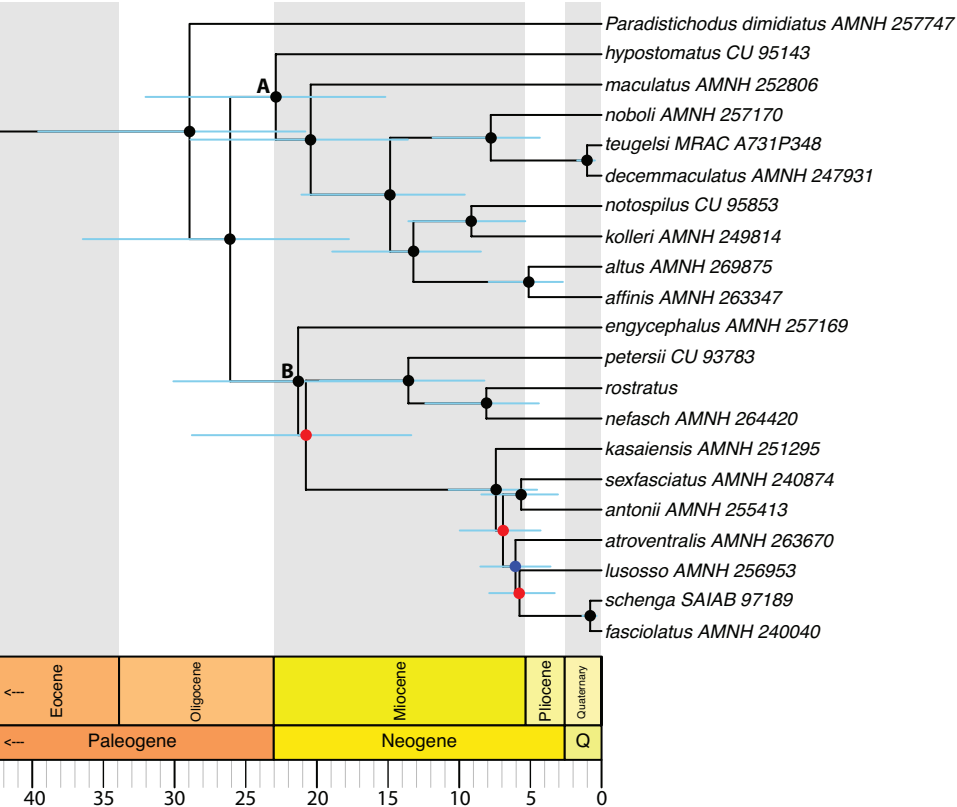

Supplement: Supplementary file 11 — Additional file 11: Figure S11. A time-scaled phylogeny of Distichodus. Chronogram resulting from BEAST2 analysis 6. Same contextual information as in Fig. 6. [file 12862_2020_1615_MOESM11_ESM.pdf]

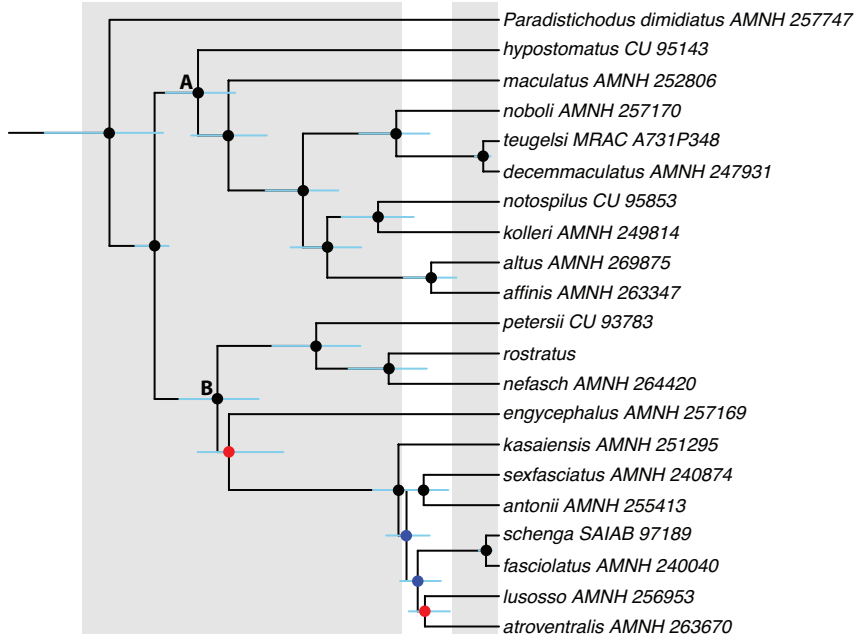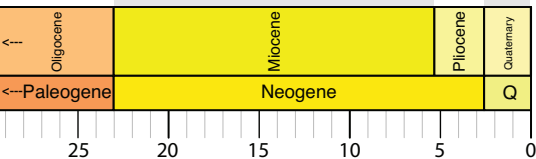

Supplement: Supplementary file 12 — Additional file 12: Figure S12. A time-scaled phylogeny of Distichodus. Chronogram resulting from BEAST2 analysis 7. Same contextual information as in Fig. 6. [file 12862_2020_1615_MOESM12_ESM.pdf]

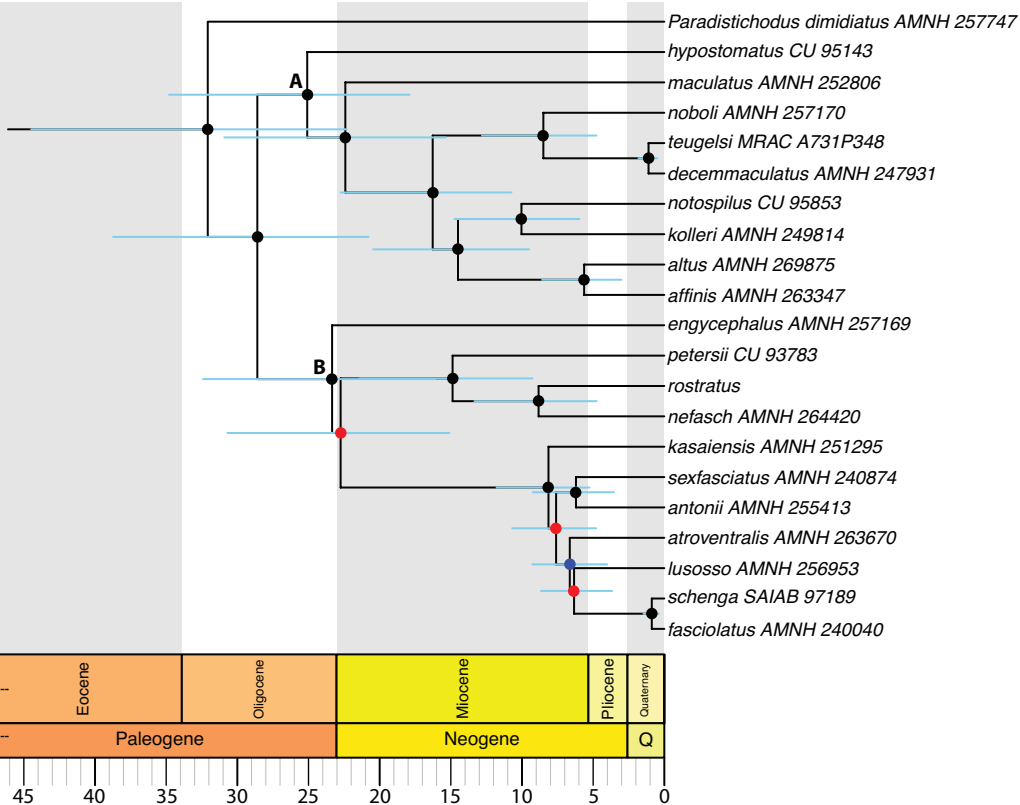

Supplement: Supplementary file 13 — Additional file 13: Figure S13. A time-scaled phylogeny of Distichodus. Chronogram resulting from BEAST2 analysis 9. Same contextual information as in Fig. 6. [file 12862_2020_1615_MOESM13_ESM.pdf]

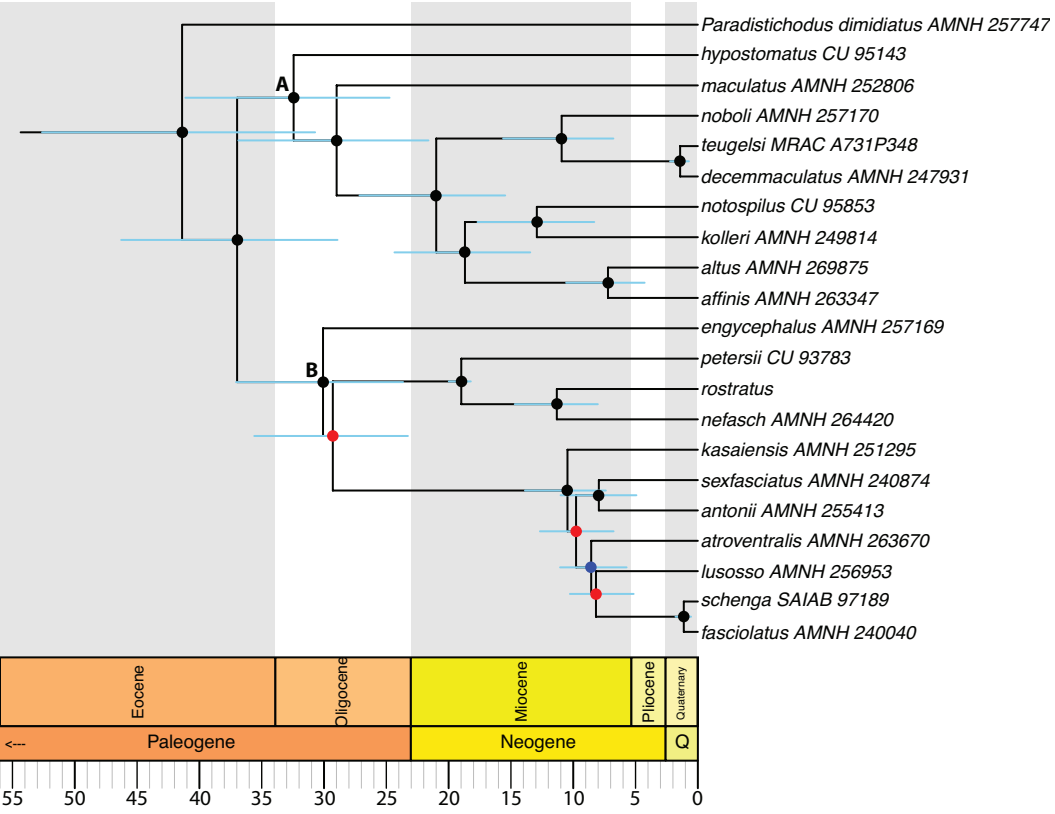

Supplement: Supplementary file 14 — Additional file 14: Figure S14. A time-scaled phylogeny of Distichodus. Chronogram resulting from BEAST2 analysis 10. Same contextual information as in Fig. 6. [file 12862_2020_1615_MOESM14_ESM.pdf]

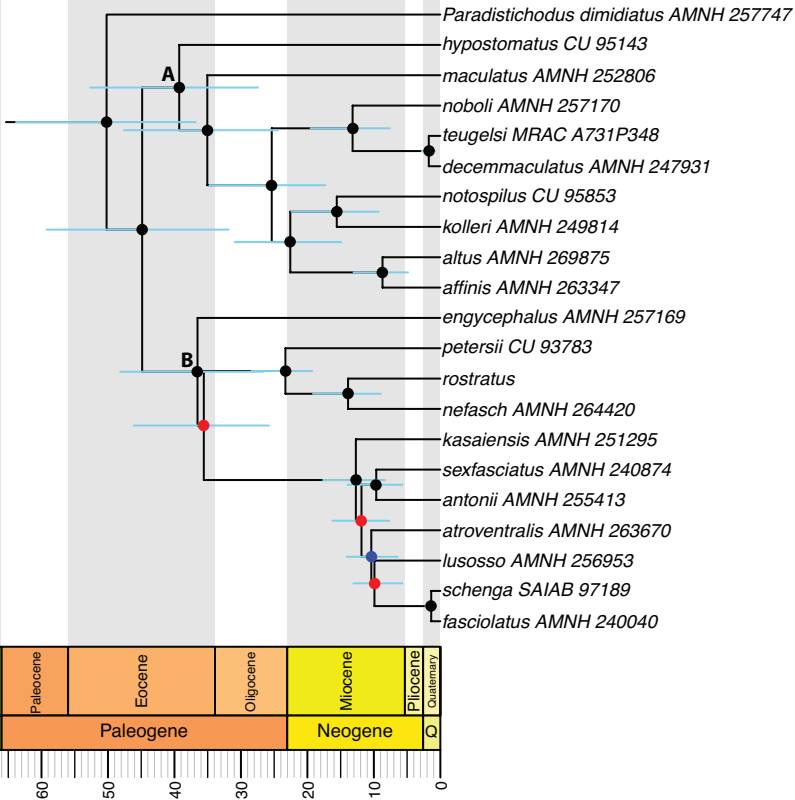

Supplement: Supplementary file 15 — Additional file 15: Figure S15. A time-scaled phylogeny of Distichodus. Chronogram resulting from BEAST2 analysis 11. Same contextual information as in Fig. 6. [file 12862_2020_1615_MOESM15_ESM.pdf]

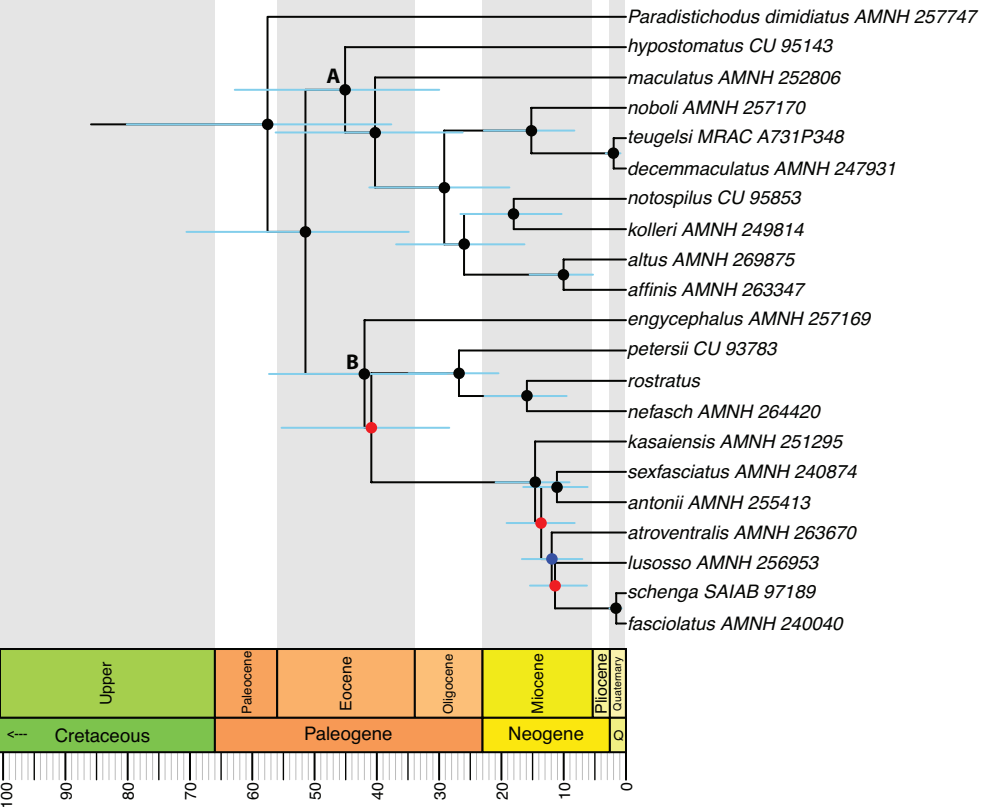

Supplement: Supplementary file 16 — Additional file 16: Figure S16. A time-scaled phylogeny of Distichodus. Chronogram resulting from BEAST2 analysis 12. Same contextual information as in Fig. 6. [file 12862_2020_1615_MOESM16_ESM.pdf]

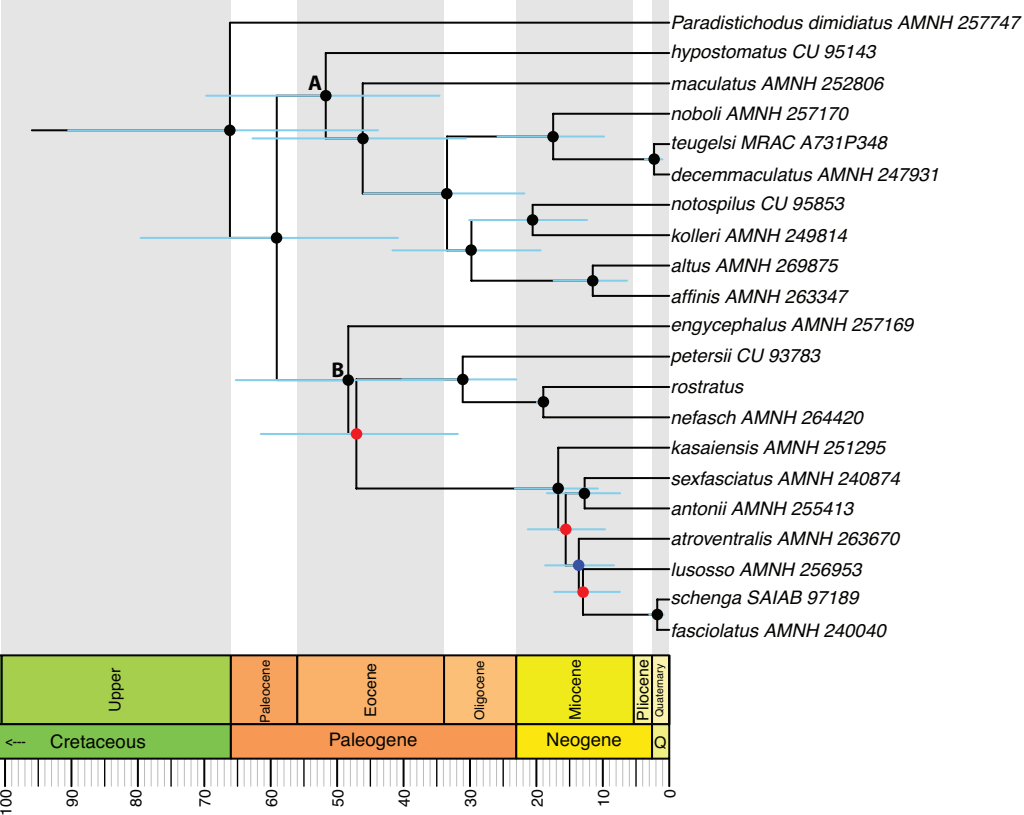

Supplement: Supplementary file 17 — Additional file 17: Figure S17. A time-scaled phylogeny of Distichodus. Chronogram resulting from BEAST2 analysis 13. Same contextual information as in Fig. 6. [file 12862_2020_1615_MOESM17_ESM.pdf]

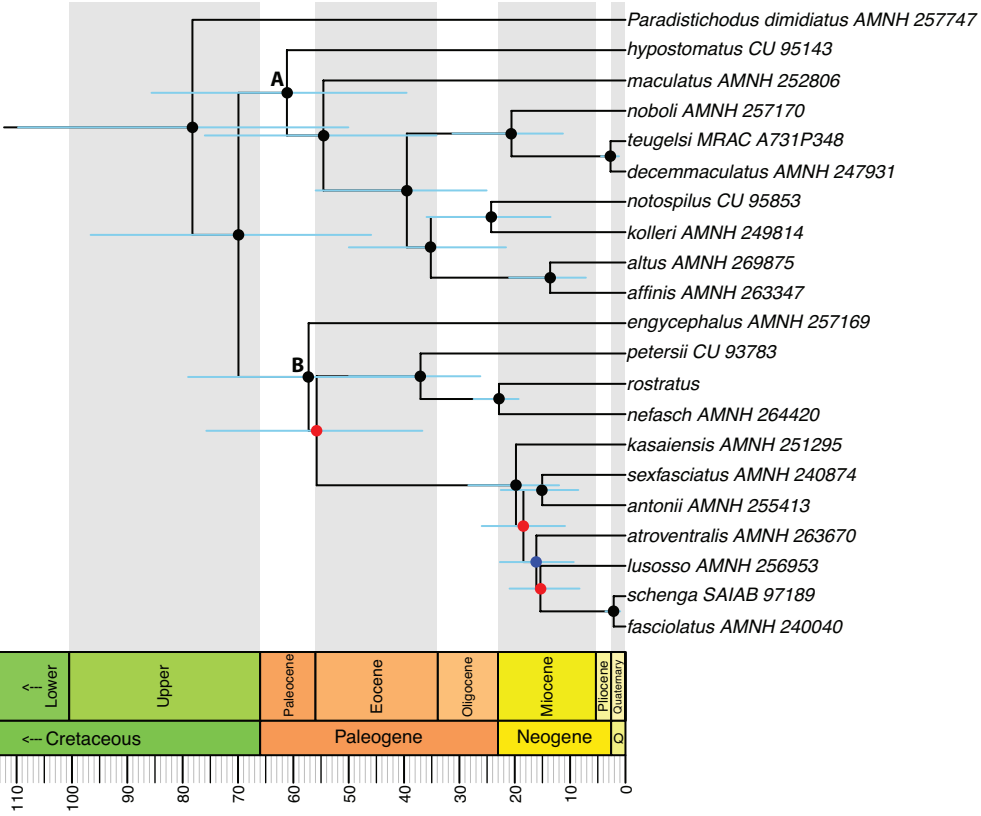

Supplement: Supplementary file 18 — Additional file 18: Figure S18. A time-scaled phylogeny of Distichodus. Chronogram resulting from BEAST2 analysis 14. Same contextual information as in Fig. 6. [file 12862_2020_1615_MOESM18_ESM.pdf]

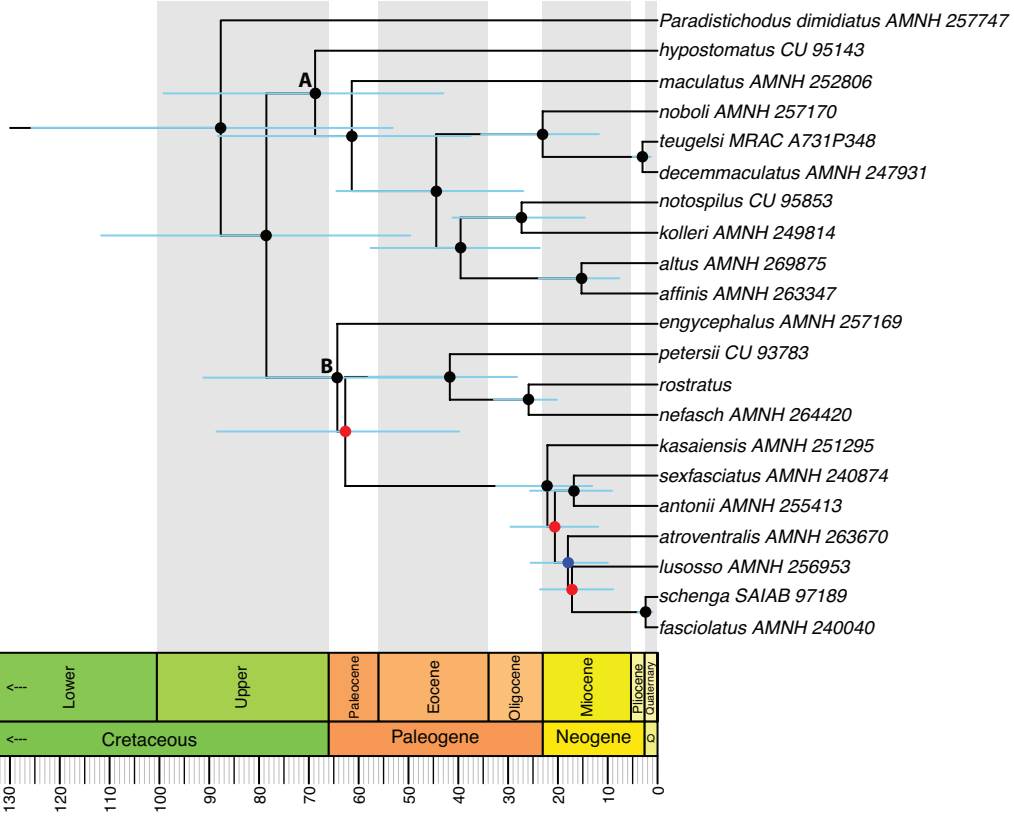

Supplement: Supplementary file 19 — Additional file 19: Fig. S19. A time-scaled phylogeny of Distichodus. Chronogram resulting from BEAST2 analysis 15. Same contextual information as in Fig. 6. [file 12862_2020_1615_MOESM19_ESM.pdf]

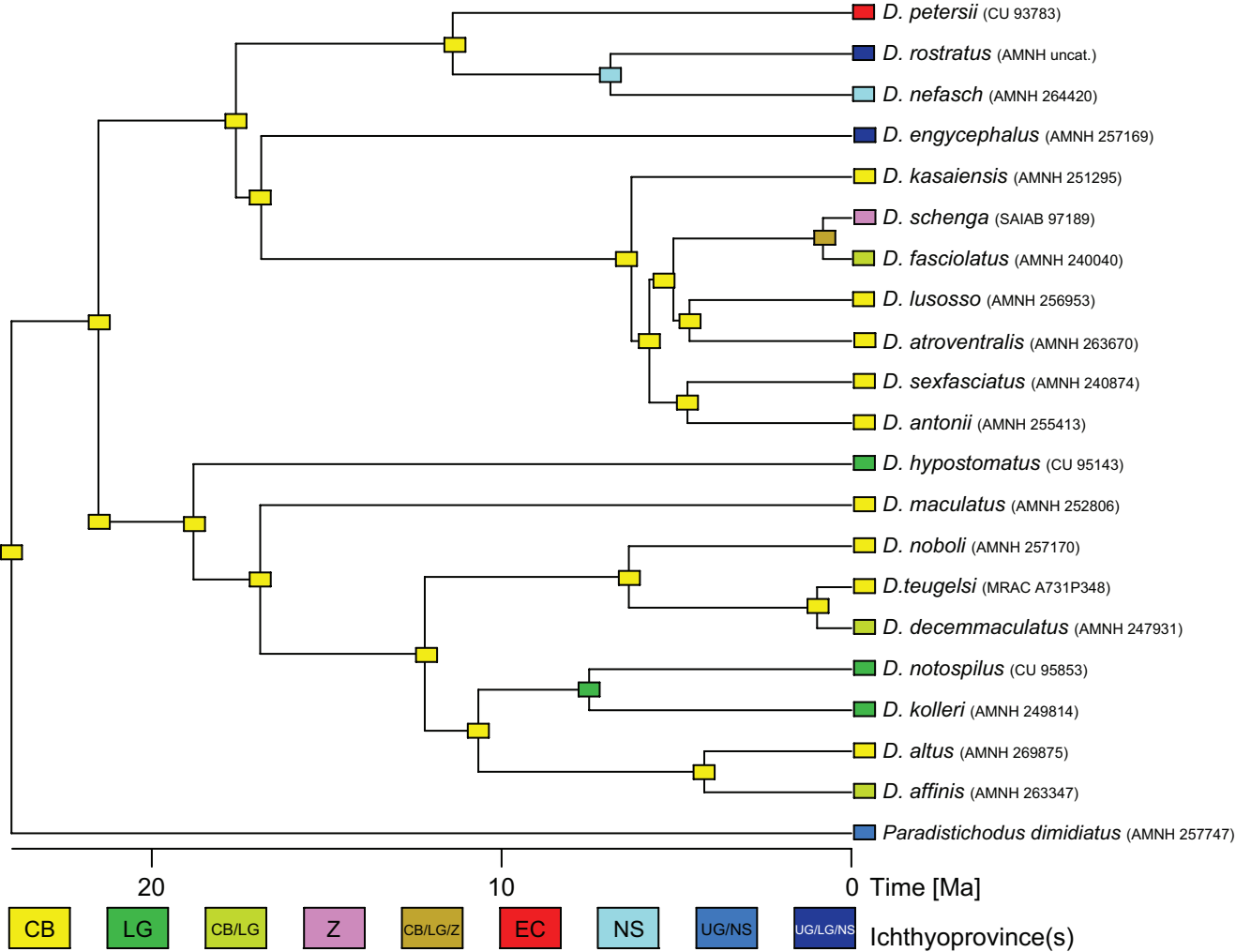

Supplement: Supplementary file 20 — Additional file 20: Figure S20. A spatiotemporal reconstruction of Distichodus range evolution. Based on the optimal DEC* model (M1; CB-as-source) and input chronogram resultant from BEAST2 analysis 5. Ichthyofaunal provinces color-coded and abbreviated as in Fig. 1. Probabilities of ancestral areas at each node are presented in Table S2. [file 12862_2020_1615_MOESM20_ESM.pdf]

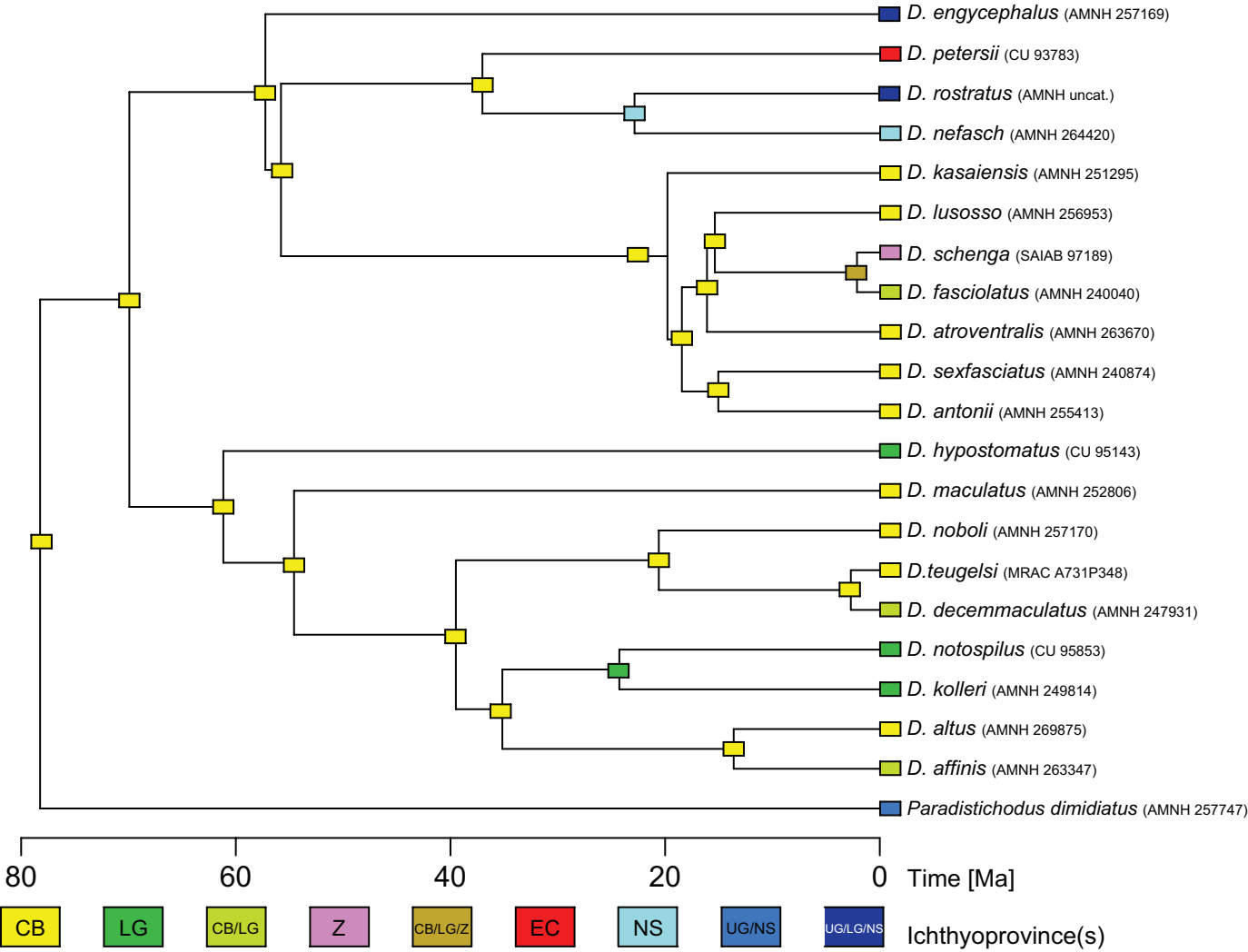

Supplement: Supplementary file 21 — Additional file 21: Figure S21. A spatiotemporal reconstruction of Distichodus range evolution. Based on the optimal DEC* model (M1; CB-as-source) and input chronogram resultant from BEAST2 analysis 14. Ichthyofaunal provinces color-coded and abbreviated as in Fig. 1. Probabilities of ancestral areas at each node are presented in Table S3. [file 12862_2020_1615_MOESM21_ESM.pdf]
